# Mechanism Integrated Information

**DOI:** 10.3390/e23030362

**Published:** 2021-03-18

**Authors:** Leonardo S. Barbosa, William Marshall, Larissa Albantakis, Giulio Tononi

**Affiliations:** 1 Department of Psychiatry, University of Wisconsin-Madison, Madison, WI 53719, USA; leonardo.barbosa@wisc.edu (L.S.B.); wmarshall3@wisc.edu (W.M.); albantakis@wisc.edu (L.A.); 2 Department of Mathematics and Statistics, Brock University, St. Catharines, ON L2S 3A1, Canada

**Keywords:** causation, consciousness, intrinsic, existence

## Abstract

The Integrated Information Theory (IIT) of consciousness starts from essential phenomenological properties, which are then translated into postulates that any physical system must satisfy in order to specify the physical substrate of consciousness. We recently introduced an information measure (Barbosa et al., 2020) that captures three postulates of IIT—existence, intrinsicality and information—and is unique. Here we show that the new measure also satisfies the remaining postulates of IIT—integration and exclusion—and create the framework that identifies maximally irreducible mechanisms. These mechanisms can then form maximally irreducible systems, which in turn will specify the physical substrate of conscious experience.

## 1. Introduction

Integrated information theory (IIT; [1,2,3]) identifies the essential properties of consciousness and postulates that a physical system accounting for it—the physical substrate of consciousness (PSC)—must exhibit these same properties in physical terms. Briefly, IIT starts from the existence of one’s own consciousness, which is immediate and indubitable. The theory then identifies five essential phenomenal properties that are immediate, indubitable and true of every conceivable experience, namely intrinsicality, composition, information, integration and exclusion. These phenomenal properties, called axioms, are translated into essential physical properties of the PSC, called postulates. The postulates are conceptualized in terms of cause–effect power and given a mathematical formulation in order to make testable predictions and allow for inferences and explanations.

So far, the mathematical formulation employed well-established measures of information, such as Kullback–Leibler divergence (KLD) [4] or earth mover’s distance (EMD) [3]. Ultimately, however, IIT requires a measure that is based on the postulates of the theory and is unique, because the quantity and quality of consciousness are what they are and cannot vary with the measure chosen. Recently, we introduced an information measure, called intrinsic difference [5], which captures three postulates of IIT—existence, intrinsicality and information—and is unique. Our primary goal here is to explore the remaining postulates of IIT—composition, integration and exclusion—in light of this unique measure, focusing on the assessment of integrated information φ for the mechanisms of a system. In doing so, we will also revisit the way of performing partitions.

The plan of the paper is as follows. In Section 2, we briefly introduce the axioms and postulates of IIT; in Section 3, we introduce the mathematical framework for measuring φ based on intrinsic difference (ID), which satisfies the postulates of IIT and is unique; in Section 4, we explore the behavior of the measure in several examples; and in Section 5, we discuss the connection between the new framework, previous versions of IIT and future developments.

## 2. Axioms and Postulates

This section summarizes the axioms of IIT and the corresponding postulates. For a complete description of the axioms and their motivation, the reader should consult [2,3,6].

Briefly, the zeroth axiom, existence, says that experience exists, immediately and indubitably. The zeroth postulate requires that the PSC must exist in physical terms. The PSC is assumed to be a system of interconnected units, such as a network of neurons. Physical existence is taken to mean that the units of the system must be able to be causally affected by or causally affect other units (take and make a difference). To demonstrate that a unit has a potential cause, one can observe whether the unit’s state can be caused by manipulating its input, while to demonstrate that a unit has a potential effect one can manipulate the state of the unit and observe if it causes the state of some other unit [7].

The first axiom, intrinsicality, says that experience is subjective, existing from the intrinsic perspective of the subject of experience. The corresponding postulate requires that a PSC has potential causes and effects within itself.

The second axiom, composition, says that experience is structured, being composed of phenomenal distinctions bound by phenomenal relations. The corresponding postulate requires that a PSC, too, must be structured, being composed by causal distinctions specified by subsets of units (mechanisms) over subsets of units (cause and effect purviews) and by causal relations that bind together causes and effects overlapping over the same units. The purviews are then subset of units whose states are constrained by another subset of units, the mechanisms, in its particular state. The set of all causal distinctions and relations within a system compose its cause–effect structure.

The third axiom, information, says that experience is specific, being the particular way it is, rather than generic. The corresponding postulate states that a PSC must specify a cause–effect structure composed of distinctions and relations that specify particular cause and effect states.

The fourth axiom, integration, says that experience is unified, in that it cannot be subdivided into parts that are experienced separately. The corresponding postulate states that a PSC must specify a cause–effect structure that is unified, being irreducible to the cause–effect structures specified by causally independent subsystems. Integrated information (Φ) is a measure of the irreducibility of the cause–effect structure specified by a system [8]. The degree Φ to which a system is irreducible can be interpreted as a measure of its existence. Mechanism integrated information (φ) is an analogous measure that quantifies the existence of a mechanism within a system. Only mechanisms that exist within a system (φ>0) contribute to its cause–effect structure.

Finally, the exclusion axiom says that experience is definite, in that it contains what it contains, neither less nor more. The corresponding postulate states that the cause–effect structure specified by a PSC should be definite: it must specify a definite set of distinctions and relations over a definite set of units, neither less nor more. The PSC and associated cause–effect structure is given by the set of units for which the value of Φ is maximal, and its distinctions and relations corresponding to maxima of φ. According to IIT, then, a system is a PSC if it is a maximum of integrated information, meaning that it has higher integrated information than any overlapping systems [3,9]. Moreover, the cause–effect structure specified by the PSC is identical to the subjective quality of the experience [10].

## 3. Theory

We first describe the process for measuring the integrated information (φ) of a mechanism based on the postulates of IIT. In order to contribute to experience, a mechanism must satisfy the postulates described in Section 2 (note that mechanisms cannot be compositional because, as components of the cause–effect structure, they cannot have components themselves). We then present some theoretical developments related to partitioning a mechanism in order to assess integration and to measuring the difference between probability distributions for quantifying intrinsic information. The subsequent process of measuring the integrated information of the system (Φ) will be discussed elsewhere.

### 3.1. Mechanism Integrated Information

**Figure 1:**
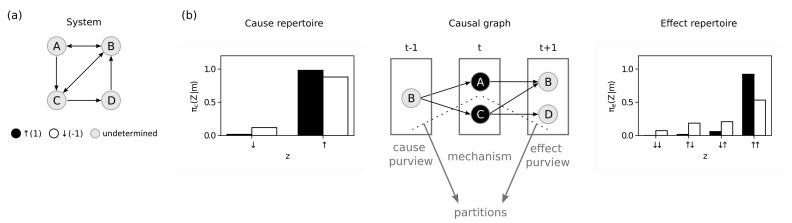
Theory. (**a**) System *S* with four random variables. (**b**) Example of a mechanism M={A,C} in state m={↑,↑} constraining a cause purview Z={B} and an effect purview Z={B,D}. Dashed lines show the partitions. The bar plots show the probability distributions, that is the cause repertoire (left) and effect repertoire (right). The black bars show the probabilities when the mechanism is constraining the purview, and the white bars show the probabilities after partitioning the mechanism.

Our starting point is a stochastic system $S=\{S_{1},S_{2},\ldots ,S_{n}\}$ with state space $\Omega_{S}$ and current state $s_{t}\in \Omega_{S}$ (Figure 1a). The system is constituted of *n* random variables that represent the units of a physical system and has a transition probability function

(1) $$p\left(s_{t+1}|s_{t}\right)=P\left(S_{t+1}=s_{t+1}|S_{t}=s_{t}\right),\;s_{t},s_{t+1}\in \Omega_{S},$$

which describes how the system updates its state (see Appendix A.1 for details). The goal is to define the integrated information of a mechanism M⊆S in a state $m_{t}\in \Omega_{M}$ based on the postulates of IIT. To this end, we will develop a difference measure $\varphi (m_{t},Z_{t\pm 1},\psi )$ which quantifies how much a mechanism *M* in state $m_{t}$ constrains the state of a purview, a set of units $Z_{t\pm 1}\subseteq S$, compared to a partition

(2) $$\psi =\{\left(M_{1},Z_{1}\right),\left(M_{2},Z_{2}\right),\ldots ,\left(M_{k},Z_{k}\right)\},$$

of the mechanism and purview into *k* independent parts (Figure 1b). As we evaluate the IIT postulates step by step, we will provide mathematical definitions for the required quantities, introduce constraints on φ and eventually arrive at a unique measure. Since potential causes of $M=m_{t}$ are always inputs to *M*, and potential effects of $M=m_{t}$ are always outputs of *M*, we will omit the corresponding update indices (t−1, *t*, t+1) unless necessary.

#### 3.1.1. Existence

For a mechanism to exist in a physical sense, it must be possible for something to change its state, and it must be able to change the state of something (it has potential causes and effects). To evaluate these potential causes and effects, we define the cause repertoire $\pi_{c}\left(Z|m\right)$ (see Equation (A2)) and the effect repertoire $\pi_{e}\left(Z|m\right)$ (see Equation (A1)), which describe how *m* constrains the potential input or output states of Z⊆S respectively (Figure 1b) [3,11,12,13].

The cause and effect repertoires are probability distributions derived from the system’s transition probability function (Equation (1)) by conditioning on the state of the mechanism and *causally marginalizing* the variables outside the purview (S\Z). Causal marginalization is also used to remove any contributions to the repertoire from units outside the mechanism (S\M). In this way, we capture the constraints due to the mechanism in its state and nothing else. Note that the cause and effect repertoires generally differ from the corresponding conditional probability distributions.

Having introduced cause and effect repertoires, we can write the difference

$$\varphi_{e}\left(m,Z,\psi \right)=D\left(\pi_{e}\left(Z|m\right),\pi_{e}^{\psi }\left(Z|m\right)\right),$$

where $\pi_{e}^{\psi }\left(Z|m\right)$ corresponds to the partitioned effect repertoire (see Equation (A3)) in which certain connections from *M* to *Z* are severed (causally marginalized). When there is no change after the partition, we require that

$$\varphi_{e}\left(m,Z,\psi \right)=0.$$

The same analysis holds for causes, replacing $\pi_{e}$ with $\pi_{c}$ in the definition of $\varphi_{c}\left(m,Z,\psi \right)$. Unless otherwise specified, in what follows we focus on effects.

#### 3.1.2. Intrinsicality

The intrinsicality postulate states that, from the intrinsic perspective of the mechanism M=m over a purview *Z*, the effect repertoire $\pi_{e}\left(Z|m\right)$ is set and has to be taken *as is*. This means that, given the purview units and their connections to the mechanism, the constraints due to the mechanism are defined by how all its units at a particular state *m* at *t* constrain all units in the effect purview at t+1 and cause purview at t−1. For example, if the mechanism fully constrains all of its purview units except for one unit which remains fully unconstrained, the mechanism cannot just ignore the unconstrained unit or optimize its overall constraints by giving more weight to some states than others in the effect repertoire. For this reason, the intrinsicality postulate should make the difference measure *D* between the partitioned and unpartitioned repertoire sensitive to a tradeoff between “expansion” and “dilution”: the measure should increase if the purview includes more units that are highly constrained by the mechanism but decrease if the purview includes units that are weakly constrained. The mathematical formulation of this requirement is given in Section 3.3.

#### 3.1.3. Information

The information postulate states that a mechanism *M*, by being in its particular state *m*, must have a specific effect, which means that it must specify a particular effect state *z* over the purview *Z*. The effect state should be the one for which *m* makes the most difference. To that end, we require a difference measure of the form

$$\varphi_{e}\left(m,Z,\psi \right)=D\left(\pi_{e}\left(Z|m\right),\pi_{e}^{\psi }\left(Z|m\right)\right)=\max_{z\in \Omega_{Z}}\left|f\left(\pi_{e}\left(z|m\right),\pi_{e}^{\psi }\left(z|m\right)\right)\right|,$$

such that the difference *D* between effect repertoires is evaluated as the maximum of the absolute value of some function *f* that is assessed for particular states. The function *f* is one of the main developments of the current work and is discussed in Section 3.3.

#### 3.1.4. Integration

The integration postulate states that a mechanism must be unitary, being irreducible to independent parts. By comparing the effect repertoire $\pi_{e}\left(Z|m\right)$ against the partitioned repertoire $\pi_{e}^{\psi }\left(Z|m\right)$, we can assess how much of a difference the partition ψ makes to the effect of *m*. To quantify how irreducible *m*’s effect is on *Z*, one must compare all possible partitioned repertoires to the unpartitioned effect repertoire. In other words, one must evaluate each possible partition ψ. Of all partitions, we define the minimum information partition (MIP)

$$\psi^{*}=\underset{\psi }{\mathrm{argmin}}\varphi_{e}\left(m,Z,\psi \right),$$

which is the one that makes the least difference to the effect. The intrinsic integrated effect information (or integrated effect information for short) of the mechanism *M* in state *m* about a purview *Z* is then defined as

$$\varphi_{e}\left(m,Z\right)=\varphi_{e}\left(m,Z,\psi^{*}\right).$$

If $\varphi_{e}\left(m,Z\right)=0$, there is a partition of the candidate mechanism that does not make a difference, which means that the candidate mechanism is reducible.

#### 3.1.5. Exclusion

The exclusion postulate states that a mechanism must be definite, it must specify a definite effect over a definite set of units. That is, a mechanism must be about a maximally irreducible purview

$$Z_{e}^{*}=\underset{Z\subseteq S}{\mathrm{argmax}}\varphi_{e}\left(m,Z\right),$$

which maximizes integrated effect information and is in the effect state

$$z_{e}^{*}=\underset{z\in \Omega_{Z_{e}^{*}}}{\mathrm{argmax}}\left|f\left(\pi_{e}\left(z|m\right),\pi_{e}^{\psi^{*}}\left(z|m\right)\right)\right|.$$

The purview $Z_{e}^{*}$ is then used to define the integrated effect information of the mechanism *M*

$$\varphi_{e}\left(m\right)=\varphi_{e}\left(m,Z_{e}^{*}\right).$$

Returning to the existence postulate, a mechanism must have both a cause and an effect. By an analogous process using cause repertoires $\pi_{c}$ instead of effect repertoires $\pi_{e}$, we can define the integrated cause information of *m*

$$\varphi_{c}\left(m\right)=\varphi_{c}\left(m,Z_{c}^{*}\right),$$

and the integrated information of the mechanism

(3) $$\varphi \left(m\right)=\min\left\{\varphi_{c}\left(m\right),\varphi_{e}\left(m\right)\right\}.$$

Thus, if a candidate mechanism *M* in state *m* is reducible over every purview either on the cause *or* effect side, φ(m)=0 and *M* does not contribute to experience. Otherwise, M=m is irreducible and forms a mechanism within the system. As such, it specifies a *distinction*

$$X\left(m\right)=\left\{\left(Z_{c}^{*}=z_{c}^{*},\;Z_{e}^{*}=z_{e}^{*},\;\varphi \left(m\right)\right):\;Z_{c}^{*},Z_{e}^{*}\subseteq S,\;z_{c}^{*}\in \Omega_{Z_{c}^{*}},\;z_{e}^{*}\in \Omega_{Z_{e}^{*}}\right\},$$

which links its maximally irreducible cause with its maximally irreducible effect, for M⊆S, $m\in \Omega_{M}$ and φ(m)∈{x∈R:x>0}. While a mechanism always specifies a unique φ(m) value, due to symmetries in the system it is possible that there are multiple equivalent solutions for $Z_{c}^{*}=z_{c}^{*}$ or $Z_{e}^{*}=z_{e}^{*}$. We expect such “ties” to be exceedingly rare in physical systems with variable connection strengths, as well as a certain amount of indeterminism and outline possible solutions to resolves “ties” in the discussion, Section 5.

### 3.2. Disintegrating Partitions

According to the integration postulate, a mechanism can only exist from the intrinsic perspective of a system if it is irreducible, meaning that any partition of the mechanism would make a difference to its potential cause or effect. Accordingly, computing the integrated information of a mechanism requires partitioning the mechanism and assessing the difference between partitioned and unpartitioned repertoires. In this section we give additional mathematical details and theoretical considerations for how to partition a mechanism together with its purview *Z*.

Generally, a partition ψ of a mechanism *M* and a purview *Z* is a set of parts as defined in Equation (2), with some restrictions on $\left(M_{i},Z_{i}\right)$. The partition "cuts apart" the mechanism, severing any connections from $M_{i}$ to $Z_{j}$ (i≠j). We use causal marginalization (see Appendix A) to remove any causal power $M_{i}$ has over $Z_{j}$ (i≠j) and compute a partitioned repertoire. Practically, it is as though we do not condition on the state of $M_{i}$ when consider $Z_{j}$. Before describing the restrictions on $\left(M_{i},Z_{i}\right)$ we will look at a few examples to highlight the conceptual issues. First, consider a third-order mechanism M={A,B,C} with the same units (as inputs or outputs) in the corresponding third order purview Z={A,B,C}. A standard example of a partition of this mechanism is

$$\psi^{1}=\left\{\left(\left\{A,B\right\},\left\{A,B\right\}\right),\left(\left\{C\right\},\left\{C\right\}\right)\right\},$$

which cuts units {A,B} away from unit {C}. Now consider the situation where we would like to additionally cut {B} in the purview away from {A,B} in the mechanism. This partition can be represented as

$$\psi^{2}=\left\{\left(\left\{A,B\right\},\left\{A\right\}\right),\left(\left\{\emptyset \right\},\left\{B\right\}\right),\left(\left\{C\right\},\left\{C\right\}\right)\right\}.$$

This example raises the issue of whether to allow the empty set as part of a partition. The question is not only conceptual but also practical, in a situation where {A,B} and {C} have opposite effects (e.g., excitatory and inhibitory connections), then it may be that the MIP $\psi^{*}=\psi^{2}$ (see Section 4.2 for an example). Here, the mechanism is always partitioned together with a purview subset.

While the definition of ψ should include partitions such as $\psi^{2}$ above, this raises additional issues. Consider the partition

$$\psi^{3}=\left\{\left(\left\{A,B,C\right\},\left\{A,B\right\}\right),\left(\left\{\emptyset \right\},\left\{C\right\}\right)\right\}.$$

In $\psi^{3}$, the set of all mechanism units is contained in one part. Should such a partition count as "cutting apart" the mechanism? The same problem arises for partitions of first-order mechanisms. Consider, for example, M={A} with purview Z={A,B,C} and partition

$$\psi^{4}=\left\{\left(\left\{A\right\},\left\{A,B\right\}\right),\left(\left\{\emptyset \right\},\left\{C\right\}\right)\right\}.$$

A first-order mechanism should be considered completely irreducible by definition, yet for the proposed partition only a small fraction of its constraint is considered integrated information: while M=A may constrain *A*, *B*, and *C*, only its constraints over *C* would be evaluated by $\psi^{4}$. A similar argument applies to $\psi^{3}$, which would only allow us to evaluate the constraint of the mechanism M={A,B,C} on *C*, not the entire purview Z={A,B,C}. In sum, $\psi^{3}$ and $\psi^{4}$ should not be permissible partitions by the integration postulate. The set of mechanism units may not remain integrated over a purview subset once a partition is applied.

Based on the above argument, we propose a set of *disintegrating partitions*

(4) $$\begin{array}{c} \Psi \left(M,Z\right)=\{{\left\{\left(M_{i},Z_{i}\right)\right\}}_{i=1}^{k}|k\in \left\{2,3,4,\ldots \right\},M_{i}\in P\left(M\right),Z_{i}\in P\left(Z\right),\\ ⋃M_{i}=M,⋃Z_{i}=Z,Z_{i}\cap Z_{j}=M_{i}\cap M_{j}=\emptyset \;\;\mathrm{for}\;\mathrm{all}\;\;i\neq j,M_{i}=M⟹Z_{i}=\emptyset \}, \end{array}$$

such that for each ψ∈Ψ(M,Z): $\left\{M_{i}\right\}$ is a partition of *M* and $\left\{Z_{i}\right\}$ is a partition of *Z* but allows the empty set to be used as a part. Moreover, if the mechanism is not partitioned into at least two parts, then the mechanism must be cut away from the entire purview.

In summary, the above definition of possible partitions ensures that the mechanism set must be divided into at least two parts, except for the special case where one part contains the whole mechanism but no units in the purview (complete partition, $\psi^{0}$). This special partition can be interpreted as “destroying” the whole mechanism at once and observing the impact its absence has on the purview.

### 3.3. Intrinsic Difference (ID)

In this section we define the measure *D*, which quantifies the difference between the unpartitioned and partitioned repertoires specified by a mechanism and thus plays an important role in measuring integrated information. We propose a set of properties that *D* should satisfy based on the postulates of IIT described above, and then identify the unique measure that satisfies them.

Our desired properties are described in terms of discrete probability distributions $P^{n}=\left[p_{1},p_{2},\ldots ,p_{n}\right]$ and $Q^{n}=\left[q_{1},q_{2},\ldots ,q_{n}\right]$. Generally, $P^{n}$ represents the cause or effect repertoire of a mechanism π(Z|m), while $Q^{n}$ represents the partitioned repertoire $\pi^{\psi }\left(Z|m\right)$.

The first property, *causality*, captures the requirement for physical existence (Section 3.1.1) that a mechanism has a potential cause and effect,

(5) $$D\left(P^{n},Q^{n}\right)=0⟺P^{n}\equiv Q^{n}.$$

The interpretation is that the integrated information *m* specifies about *Z* is only zero if the unpartitioned and partitioned repertoires are identical. In other words, by being in state *m*, the mechanism *M* does not constrain the potential state of *Z* above its partition into independent parts.

The second property, *intrinsicality*, captures the requirement that physical existence must be assessed from the perspective of the mechanism itself (Section 3.1.2). The idea is that information should be measured from the intrinsic perspective of the candidate mechanism *M* in state *m*, which determines the potential state of the purview *Z* by itself, independent of external observers. In other words, the constraint *m* has over *Z* must depend only on their units and connections. In contrast, traditional information measures were conceived to quantify the amount of signal transmitted across a channel between a sender and a receiver from an extrinsic perspective, typically that of a channel designer who has the ability to optimize the channel’s capacity. This can be done by adjusting the mapping between the states of *M* and *Z* through encoders and decoders to reduce indeterminism in the signal transmission. However, such a remapping would require more than just the units and connections present in *M* and *Z*, thus violating intrinsicality [5].

The intrinsicality property is defined based on the behavior of the difference measure when distributions are extended by adding units to the purview or increasing the number of possible states of a unit [14]. A distribution $P_{1}^{n}$ is extended by a distribution $P_{2}^{n}$ to create a new distribution $P_{1}^{n}⊗P_{2}^{n}$, where ⊗ is the Kronecker product. When a fully selective distribution (one where an outcome occurs with probability one) is extended by another fully selective distribution, the measure should increase additively (expansion). However, if a distribution is extended by a fully undetermined distribution (one where all *n* outcomes are equally likely), then the measure should decrease by a factor of *n* (dilution). For expansion, suppose $P_{1}^{n}$ and $P_{2}^{n}$ are fully selective distributions, then for any $Q_{1}^{n}$ and $Q_{2}^{n}$ we have

(6) $$D\left(P_{1}^{n}⊗P_{2}^{n},Q_{1}^{n}⊗Q_{2}^{n}\right)=D\left(P_{1}^{n},Q_{1}^{n}\right)+D\left(P_{2}^{n},Q_{2}^{n}\right).$$

For dilution, suppose $P_{2}^{n}$ and $Q_{2}^{n}$ are fully undetermined distributions, then for any $P_{1}^{n}$, $Q_{1}^{n}$ we have

(7) $$D\left(P_{1}^{n}⊗P_{2}^{n},Q_{1}^{n}⊗Q_{2}^{n}\right)=\frac{1}{n}D\left(P_{1}^{n},Q_{1}^{n}\right).$$

Together, Equations (6) and (7) define the intrinsicality property.

The final property, *specificity*, requires that physical existence must be about a specific purview state (Section 3.1.3),

(8) $$D\left(P^{n},Q^{n}\right)=\max_{\alpha }\left|f(p_{\alpha },q_{\alpha })\right|.$$

The function f(p,q) defines the difference between two probability distributions at a specific state of the purview. The mechanism is defined based on the state that maximizes its difference within the system.

Previous work employed similar properties to quantify intrinsic information but used a version of the specificity property that did not include the absolute value [5]. In that work, the goal was to compute the intrinsic information of a communication channel, with an implicit assumption that the source is sending a specific message. In that context, a signal is only informative if it increases the probability of receiving the correct message. Here we are interested in integrated information within the context of the postulates of IIT as a means to quantify existence, which requires causes and effects. A mechanism can be seen as having an effect (or cause) whether it increases or decreases the probability of a specific state.

Together, the three properties (causality, specificity, and intrinsicality) characterize a unique measure, the intrinsic difference, for measuring the integrated information of a mechanism. Note that while causality (Equation (5)) and expansion (Equation (6)) properties are traditionally required by information measures (see [15]), here we also require dilution (Equation (7)) and specificity (Equation (8)). While the maximum operation present in specificity in order to select one specific purview state seems to us uncontroversial, one may argue that the dilution factor $\frac{1}{n}$ in Equation (7) is somewhat arbitrary. However, note that if specificity requires that information is specific to one state, after adding a fully undetermined distribution of size *n* to the purview, the amount of causal power measured by the function *f* in state α will be invariably divided by *n*. This way, we believe that the dilution factor must be necessarily $\frac{1}{n}$, at least in this particular case.

**Theorem** **1.** *If $D(P^{n},Q^{n})$ satisfies the*causality*,*intrinsicality*, and*specificity*properties, then*

$$D\left(P^{n},Q^{n}\right)=\max_{\alpha }\left|f(p_{\alpha },q_{\alpha })\right|,$$

*where*

$$f\left(p,q\right)=k\;p\log\left(\frac{p}{q}\right).$$

The full mathematical statement of the theorem and its proof are presented in Appendix B. For the rest of the manuscript we assume k=1 without loss of generality. Here, our main interest is using ID to quantify the difference between unpartitioned and partitioned cause or effect repertoires when assessing the integrated information of a mechanism,

$$\varphi \left(m,Z\right)=D\left(\pi \left(Z|m\right),\pi^{\psi^{*}}\left(Z|m\right)\right)=\max_{z\in \Omega_{Z}}\left|\pi \left(z|m\right)\log\left(\frac{\pi (z|m)}{\pi^{\psi^{*}}\left(z|m\right)}\right)\right|.$$

One can interpret the integrated information as being composed of two terms. First, the informativeness

$$\left|\log\left(\frac{\pi (z|m)}{\pi^{\psi^{*}}\left(z|m\right)}\right)\right|,$$

which reflects the difference in Hartley information contained in state *z* before and after the partition. Second, the selectivity

π(z∣m),

which reflects the likelihood of the cause or effect. Together, the two terms can be interpreted as the density of information for a particular state [5].

## 4. Methods and Results

**Figure 2:**
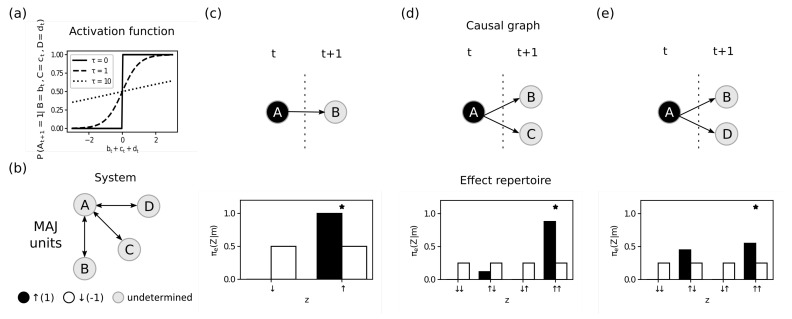
Intrinsicality. (**a**) Activation functions without bias (η=0) and different levels of constraint (τ=0, τ=1 and τ=10). (**b**) System *S* analyzed in this figure. The remaining panels show on top the causal graph of the mechanism M={A} at state m={1} constraining different output purviews and on the bottom the probability distributions of the purviews (effect repertoires). The black bars show the probabilities when the mechanism is constraining the purview, and the white bars show the unconstrained probabilities after the complete partition $\psi^{0}$. The “*” indicates the state selected by the maximum operation in the intrinsic difference (ID) function. (**c**) The mechanism fully constrains the unit *B* in the purview Z={B} ($\tau_{B}=0$), resulting in state z={↑} defining the amount of intrinsic information in the mechanism as $\varphi \left(m,Z,\psi^{0}\right)=ID(\pi_{e}(B|M=↑)|\pi_{e}^{\psi^{0}}(B|M=↑))=\pi_{e}(B=↑|A=↑)\cdot |\log(\pi_{e}\left(B=↑|A=↑\right)/\pi_{e}^{\psi^{0}}(B=↑|M=↑))|=1\cdot 0.69=0.69$. (**d**) After adding a slightly undetermined unit ($\tau_{C}=1$) to the purview (Z={B,C}), the intrinsic information increases to 1.11. The new maximum state (z={↑,↑}) has now much higher informativeness ($|\log(\pi_{e}\left(BC=↑↑|A=↑\right)/\pi_{e}^{\psi^{0}}(BC=↑↑|A=↑))|=1.26$) but only slightly lower selectivity (π(BC=↑↑|A=↑)=0.89), resulting in expansion. (**e**) When instead of *C*, we add the very undetermined unit *D* to the purview ($\tau_{D}=10$), the new purview (Z={B,D}) has a new maximum state (z={↑,↑}) with marginally higher informativeness ($|\log(\pi_{e}\left(BC=↑↑|A=↑\right)/\pi_{e}^{\psi^{0}}(BC=↑↑|A=↑))|=0.79$) and very low selectivity ($\pi_{e}\left(BC=↑↑|A=↑\right)=0.55$), resulting in dilution.

Throughout this section we investigate each step necessary to compute φ(m), the integrated information of a mechanism *M* in state *m*. To this end, we construct systems *S* formed by units A,B,C,… that are either ↑ (1) or ↓ (−1) at time *t* with probability of being ↑ defined by (Figure 2a)

(9) $$\begin{array}{c} P\left(Y_{t}=1|A_{t-1}=a_{t-1},B_{t-1}=b_{t-1},\ldots \right)=\frac{1}{1+\exp\left\{-\frac{2(a_{t-1}+b_{t-1}+\ldots +h)}{\tau }\right\}}, \end{array}$$

for all Y∈S, where A,B,… are the units that input to *Y*. Besides the sum of the input states, the function depends on two parameters: h∈R defines a bias towards being ↑ (h>0) or ↓ (h<0), while τ∈{x∈R:x≥0} defines how deterministic unit *A* is. For τ⟶∞, the unit turns ↑ or ↓ with equal probability (fully undetermined), while for τ=0 it turns ↑ whenever the sum of the inputs is greater than the threshold η, and turns ↓ otherwise (fully selective; Figure 2a). This way, τ=0 means that the unit is fully constrained by the inputs (deterministic), τ=1 means the unit is partially constrained, and τ=10 means the unit is only weakly constrained, etc. Unless otherwise specified, in the following we focus on investigating effect purviews.

### 4.1. Intrinsic Information

We start by investigating the role of intrinsicality in computing the integrated information of a mechanism. To this end, we will compare $\varphi_{e}\left(m,Z,\psi^{0}\right)$ for various mechanism-purview pairs, which evaluates the ID over a complete partition

$$\psi^{0}=\left\{\left(\left\{M\right\},\left\{\emptyset \right\}\right),\left(\left\{\emptyset \right\},\left\{Z\right\}\right)\right\}$$

of mechanism *M* and purview units *Z*, leaving the purview fully unconstrained after the partition (in this case, the partitioned repertoires are equivalent to the unconstrained repertoires defined in Equation (A5) and Equation (A4)). Intrinsicality requires that the ID must increase additively when fully constrained units are added to the purview (expansion, Equation (6)) and decrease exponentially when fully unconstrained units are added to the purview (dilution, Equation (7)). We define the system *S* depicted in Figure 2b to investigate the expansion and dilution of a mechanism M={A} over different purviews Z⊆S. Next, we fix the mechanism *M* in state m=1 and measure the ID of this mechanism over effect purviews with varying levels of indeterminism τ but a fixed threshold h=0 (partially deterministic majority gates).

First consider the purview Z={B} with a fully constrained unit ($\tau_{B}=0$), such that (Figure 2B)

$$\varphi_{e}\left(m,Z,\psi^{0}\right)=\mathrm{ID}\left(\pi_{e}\left(B|A=↑\right),\pi_{e}^{\psi^{0}}\left(B|A=↑\right)\right)=0.69.$$

Now consider the same mechanism over a larger purview Z={B,C}, which has an additional, partially constrained unit *C* ($\tau_{C}=1$). This purview has a larger repertoire of possible states, resulting in a larger difference between partitioned and unpartitioned probabilities of one state (high informativeness). At the same time, the probability of this state is still very high in absolute terms (high selectivity). Thus, the ID of *m* over {B,C} is *higher* than over {B} alone (Figure 2c):

$$\varphi_{e}\left(m,Z,\psi^{0}\right)=\mathrm{ID}\left(\pi_{e}\left(BC|A=↑\right),\pi_{e}^{\psi^{0}}\left(BC|A=↑\right)\right)=1.11.$$

The higher value for Z={B,C} reflects the *expansion* that occurs whenever informativeness increases while selectivity is still high. Notice that the expansion here is subadditive since the new unit is constrained but not *fully* constrained (or fully selective).

Finally, consider another purview Z={B,D}, where *D* is only weakly constrained ($\tau_{D}=10$). While the new purview has a state where informativeness is marginally higher than before, selectivity is much lower (the state has much lower probability). For this reason, $\varphi_{e}\left(m,Z,\psi^{0}\right)$ is lower for Z={B,D} than for the smaller purview Z={B}, reflecting *dilution* (Figure 2c):

$$\varphi_{e}\left(m,Z,\psi^{0}\right)=\mathrm{ID}\left(\pi_{e}\left(BD|A=↑\right),\pi_{e}^{\psi^{0}}\left(BD|A=↑\right)\right)=0.43.$$

Notice that dilution here is not exactly a factor of 2 since the new unit is weakly constrained by the mechanism but not *fully* unconstrained.

**Figure 3:**
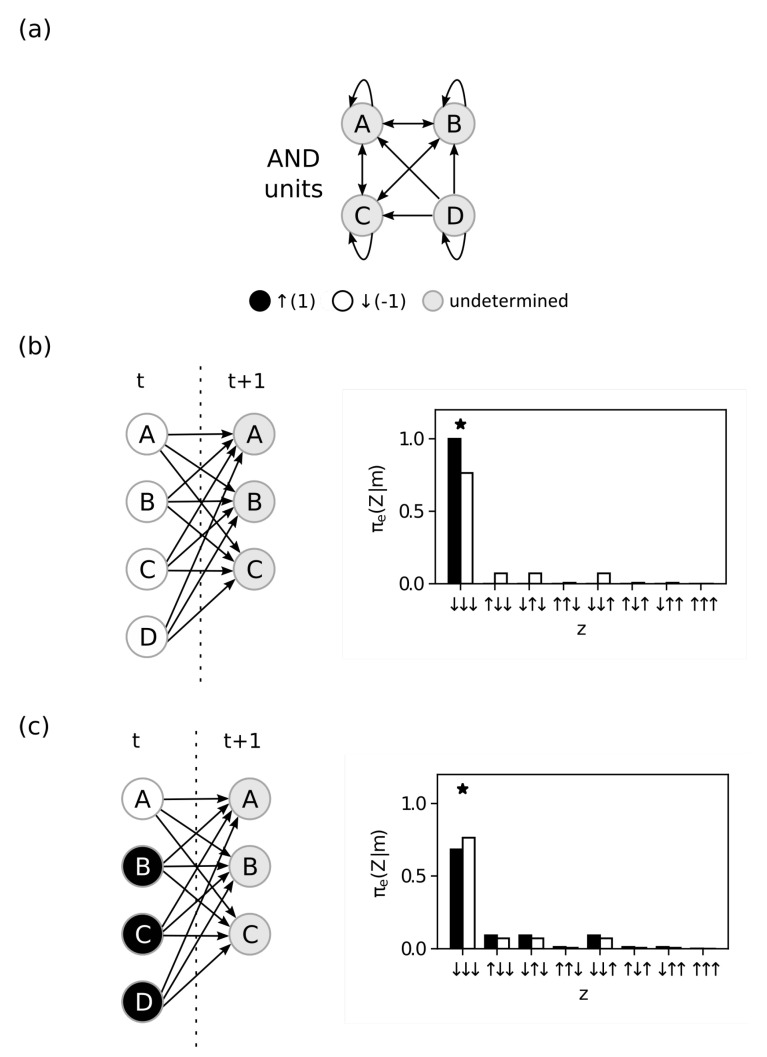
Information. (**a**) System *S* analyzed in this figure. All units have τ=1 and η=−3 (partially deterministic AND gates). The remaining panels show on the left the time unfolded graph of the mechanism M={A,B,C,D} constraining different output purviews and on the right the probability distribution of the purview Z={A,B,C} (effect repertoires). The black bars show the probabilities when the mechanism is constraining the purview, and the white bars show the unconstrained probabilities after the complete partition. The “*” indicates the state selected by the maximum operation in the ID function. (**b**) The mechanism at state m={↓,↓,↓,↓}. The purview state z={↓,↓,↓} is not only the most constrained by the mechanism (high informativeness) but also very dense (high selectivity). As a result, it has intrinsic information higher than all other states in the purview and defines the intrinsic information of the mechanism as 0.27. (**c**) If we change the mechanism state to m={↓,↑,↑,↑}, the probability of observing the purview state z={↓,↓,↓} is now *smaller* than chance. However, this probability is still very *different* from chance and therefore very constrained by the mechanism (high informativeness). At the same time, the state is still very *dense*, meaning it has a probability of happening much higher than all other states (high selectivity). Together, they define the intrinsic information of the state, which is higher than the intrinsic information of all other states in the purview, defining the intrinsic information of the mechanism as 0.08.

Next we investigate the role of the information postulate, which requires that the mechanism must be *specific*, meaning that a mechanism must both be in a specific state and specify an effect state (or a cause state) of a specific purview. Consider the system in Figure 3a where we focus on a high-order mechanism with four units M={A,B,C,D} over a purview with three units Z={A,B,C}. The threshold and amount of indeterminism of the purview units are fixed: h=−3 and τ=1, which makes the purview units function like partially deterministic AND gates. We show not only that the mechanism can be more or less informative depending on its state but also that the specific purview state selected by the ID measure depends both on the probability of the state and on how much the state is constrained by the mechanism.

When the state of the mechanism is m={↓,↓,↓,↓} (Figure 3b), the most informative state in the purview is z={↓,↓,↓} since all units are *more* likely to be turned ↓ than they are after partitioning (high informativeness), and at the same time this state still has high probability (high selectivity). Out of all states, z={↓,↓,↓} maximizes informativeness and selectivity in combination, resulting in

$$\begin{array}{cc} \varphi_{e}\left(m,Z,\psi^{0}\right) & =\mathrm{ID}(\pi_{e}\left(ABC|ABCD=↓↓↓↓\right),\pi_{e}^{\psi^{0}}\left(ABC|ABCD=↓↓↓↓)\right)\\ \; & =0.27. \end{array}$$

A different scenario is depicted if we change the state of the mechanism to ABCD={↓,↑,↑,↑} (Figure 3c). In this mechanism state the constrained probability of ABC={↓,↓,↓} is *lower* than than the probability after partitioning. However, the mechanism is informative because the probabilities are different. At the same time, the state ABC={↓,↓,↓}*still* has high probability while being constrained by the mechanism ABCD={↓,↑,↑,↑}. Together the product of the informativeness and selectivity is higher for the purview state {↓,↓,↓} than any other state, resulting in

$$\begin{array}{cc} \varphi_{e}\left(m,Z,\psi^{0}\right) & =\mathrm{ID}(\pi_{e}\left(ABC|ABCD=↓↑↑↑\right),\pi_{e}^{\psi^{0}}\left(ABC|ABCD=↓↑↑↑)\right)\\ \; & =0.08. \end{array}$$

Although it may be counterintuitive to identify an effect state whose probability is decreased by the mechanism, it highlights an important feature of intrinsic information: it balances informativeness and selectivity. Informativeness is about *constraint*, meaning how much the probability of observing a given state in the purview *changes* due to being constrained by the mechanism. At the same time, selectivity is about probability *density* at a given state, meaning that this constraint is only relevant if the state is realized by the purview. If the mechanism is informative while increasing selectivity, then there is no tension between the two. However, whenever the mechanism decreases the probability of a state, there is a tension between how informative and how selective that state is. As long as *together* the product of informativeness and selectivity of a state (in this case ABC={↓,↓,↓}) is higher than all other states, it is selected by the maximum operation in the ID function and thus determines the intrinsic information of the mechanism.

### 4.2. Integrated Information

**Figure 4:**
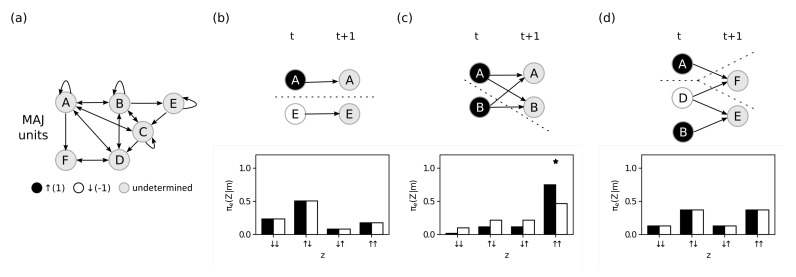
Integration. (**a**) System *S* analysed in this figure and in Figure 5. All units have τ=1 and η=0 (partially deterministic MAJORITY gates). The remaining panels show on the top the time unfolded graph of different mechanisms constraining different output purviews and on the bottom the probability distributions (effect repertoires). The black bars show the probabilities when the mechanism is constraining the purview, and the white bars show the partitioned probabilities. The “*” indicates the state selected by the maximum operation in the ID function. (**b**) The mechanism M={A,E} in state m={↑,↓} constraining the purview Z={A,E}. While the complete partition has nonzero intrinsic information, the mechanism is clearly not integrated, as revealed by the MIP partition $\psi^{*}=\left\{\left(\left\{A,\right\},\left\{A\right\}\right),\left(\left\{E,\right\},\left\{E\right\}\right)\right\}$, resulting in zero *integrated* information. (**c**) The mechanism M={A,B} in state m={↑,↑} constraining the purview Z={A,B}. The partition $\psi^{*}=\left\{\left(\left\{A,\right\},\left\{A,B\right\}\right),\left(\left\{B\right\},\left\{\emptyset \right\}\right)\right\}$ has less intrinsic information than any other partition, i.e., it is the MIP of this mechanism, and it defines the integrated information as 0.36. (**d**) The mechanism M={A,B,D} in state m={↑,↑,↓} constraining the purview Z={E,F}. The tri-partition $\psi^{*}=\left\{\left(\left\{A\right\},\left\{\emptyset \right\}\right),\left(\left\{\emptyset ,\right\},\left\{F\right\}\right),\left(\left\{B,D\right\},\left\{E\right\}\right)\right\}$ is the MIP and it shows that the mechanism is not integrated, i.e, the mechanism has zero integrated information.

The integration postulate of IIT requires that mechanisms be *integrated* or irreducible to parts. In this section we use the system defined in Figure 4a, with η=0 and τ=1 for all units, to investigate how mechanisms are impacted by different partitions. We compute the ID between the intact and all possible partitioned effect repertoires to measure the impact of each partition ψ∈Ψ(M,Z). We identify the partition with *lowest* ID as the MIP of the candidate mechanism over a purview.

First, when considering the mechanism M={A,E}={↑,↓} over the purview Z={A,E}, the complete partition $\psi^{0}$ (partitioning the entire mechanism away from the entire purview) assigns a positive value

$$\varphi_{e}\left(m,Z,\psi^{0}\right)=\mathrm{ID}(\pi_{e}\left(AE|AE=↑↓\right),\pi_{e}^{\psi^{0}}(AE|AE=↑↓))=0.36.$$

However, if we try the partition

$$\psi^{1}=\left\{\left(\left\{A\right\},\left\{A\right\}\right),\left(\left\{E\right\},\left\{E\right\}\right)\right\},$$

we find that the candidate mechanism is not integrated (as is obvious after inspecting Figure 4b):

$$\varphi_{e}\left(m,Z,\psi^{1}\right)=\mathrm{ID}(\pi_{e}\left(AE|AE=↑↓\right),\pi_{e}^{\psi^{1}}(AE|AE=↑↓))=0.$$

We conclude that this candidate mechanism does not exist within the system over this purview.

Next, we consider the candidate mechanism M={A,B}={↑,↑} over the purview Z={A,B}. We observe that the partition

$$\psi^{2}=\left\{\left(\left\{A\right\},\left\{A,B\right\}\right),\left(\left\{B\right\},\left\{\emptyset \right\}\right)\right\},$$

defines an intrinsic difference

$$\varphi_{e}\left(m,Z,\psi^{2}\right)=\mathrm{ID}(\pi_{e}\left(AB|AB=↑↑\right),\pi_{e}^{\psi^{2}}(AB|AB=↑↑))=0.36,$$

which is *smaller* than the one assigned by the complete partition $\psi^{0}$,

$$\varphi_{e}\left(m,Z,\psi^{0}\right)=\mathrm{ID}(\pi_{e}\left(AB|AB=↑↑\right),\pi_{e}^{\psi^{0}}(AB|AB=↑↑))=0.51.$$

Although the ID over $\psi^{2}$ is smaller than that over the complete partition, this information is not *zero*. Moreover, the partition $\psi^{2}$ yields an ID value that is smaller than *any* other partition ψ∈Ψ(AB,AB). In this case, we say that $\psi^{2}$ is the MIP ($\psi^{*}=\psi^{2})$, and that the candidate mechanism M={A,B} has *integrated* effect information (Figure 4c):

$$\varphi_{e}\left(m,Z\right)=\mathrm{ID}(\pi (AB|AB=↑↑)|\pi^{\psi^{*}}(AB|AB=↑↑))=0.36.$$

Finally, for the candidate mechanism M={A,B,D}={↑,↑,↓} over the purview Z={E,F}, any partition that does not include the empty set as a part in $\left\{M_{i}\right\}$ leads to nonzero ID. However, if we allow the empty set for $M_{i}$ (as discussed in Section 3.2), the candidate mechanism is reducible because disintegrating it with the partition

$$\psi^{*}=\left\{\left(\left\{A\right\},\left\{\emptyset \right\}\right),\left(\left\{\emptyset \right\},\left\{F\right\}\right),\left(\left\{B,D\right\},\left\{E\right\}\right)\right\}$$

makes no difference to the purview states, resulting in

$$\varphi_{e}\left(m,Z\right)=\mathrm{ID}(\pi_{e}\left(EF|ABD=↑↑↓\right),\pi_{e}^{\psi^{*}}(EF|ABD=↑↑↓))=0.$$

This occurs since *B* and *D* have opposite effects over the purview unit *E*, and by cutting both inputs to *E* we avoid changing the repertoire. Therefore, M={A,B,D} does not exist as a mechanism over the purview Z={E,F}.

### 4.3. Maximal Integrated Information

The last postulate we investigate is exclusion, which dictates that mechanisms are defined over a *definite* purview, the one over which the mechanism is maximally irreducible (has maximal integrated effect information). Using the system defined in Figure 4a, we investigate two candidate mechanisms. First, we study the candidate mechanism M={A}=↑, similar to the one in Figure 2. Since M={A} is first order (constituted of one unit), there is only one possible partition (the complete partition)

$$\psi^{*}=\psi^{0}=\left\{\left(\left\{A\right\},\left\{\emptyset \right\}\right),\left(\left\{\emptyset \right\},\left\{Z\right\}\right)\right\}.$$

After computing $\varphi_{e}\left(m,Z\right)$ for all possible purviews Z∈S, we find that the mechanism has maximum integrated effect information over the purview $Z_{e}^{*}=\left\{A,F\right\}$, thus according to Equation (3) we have

$$\varphi_{e}\left(m\right)=\mathrm{ID}\left(\pi_{e}\left(AF|A=↑\right),\pi_{e}^{\psi^{*}}\left(AF|A=↑\right)\right)=0.36.$$

Next, similarly to Figure 3, we investigate the candidate mechanism M={A,B,C,D}={↑,↑,↑,↓}. After computing $\varphi_{e}\left(m,Z,\psi \right)$ over all possible purviews in the system (Z⊆S) and over all possible partitions for each purview (ψ∈Ψ(ABCD,Z)), we find that the mechanism has maximum integrated effect information over the purview $Z_{e}^{*}=\left\{A,B,C\right\}$, with partition

$$\psi^{*}=\left\{\left(\left\{A,B,C\right\},\left\{A,B,C\right\}\right),\left(\left\{D\right\},\left\{\emptyset \right\}\right)\right\},$$

and that

$$\begin{array}{cc} \varphi_{e}\left(m\right) & =\mathrm{ID}(\pi_{e}\left(ABC|ABCD=↑↑↑↓\right),\pi_{e}^{\psi^{*}}\left(ABC|ABCD=↑↑↑↓)\right)\\ \; & =0.04. \end{array}$$

**Figure 6:**
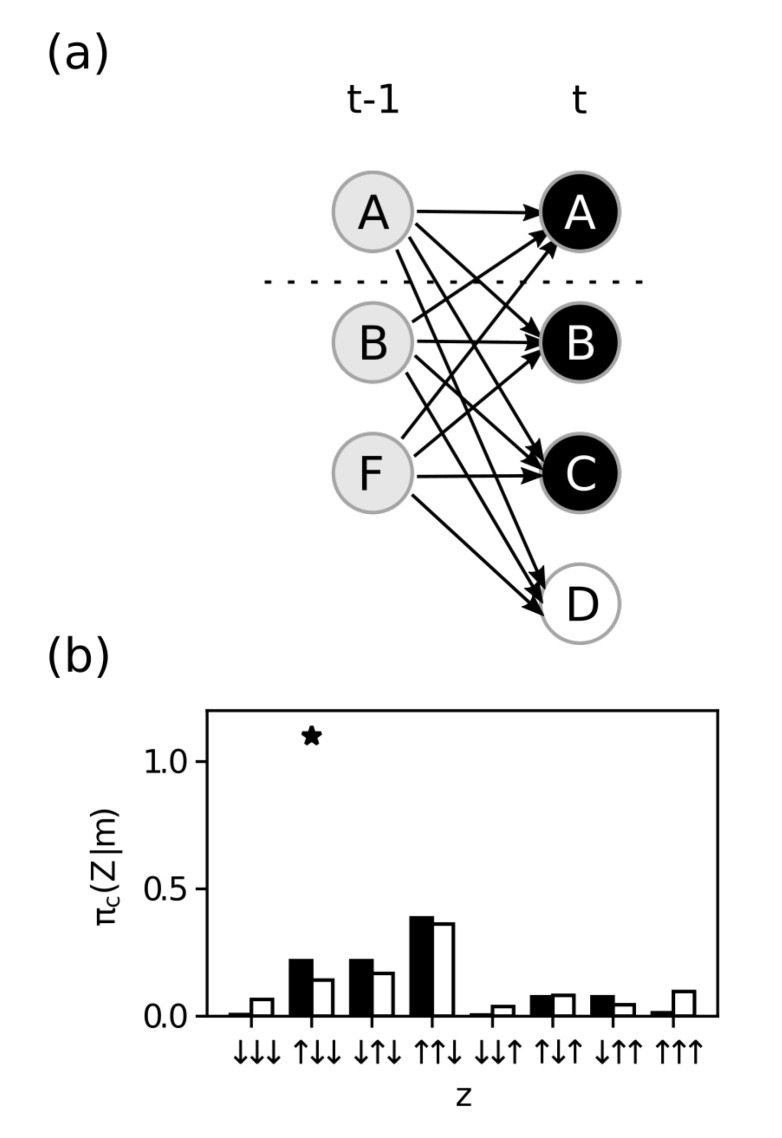
Integrated cause information. (**a**) Causal graph of mechanism M={A,B,C,D} at state m={↑,↑,↑,↓} constraining the purview Z={A,B,F}, which has the maximum integrated information of all Z⊆S and defines the mechanism integrated information. (**b**) The black bars show the probabilities when the mechanism is constraining the purview (cause repertoire), and the white bars show the probabilities after the partition (partitioned cause repertoire). The “*” indicates the state selected by the maximum operation in the ID function and defines $Z_{c}^{*}$.

Finally, IIT requires that mechanisms have *both* causes and effects within the system. We perform an analogous process using the cause repertoire $\pi_{c}\left(Z|ABCD=↑↑↑↓\right)$ (see Equation (A2) and Figure 6) to identify the maximally irreducible cause purview $Z_{c}^{*}$. We find that $Z_{c}^{*}=\left\{A,B,F\right\}$ with MIP

$$\psi^{*}=\{\left(\left\{A\right\},\left\{A\right\}\right),\left(\left\{B,C,D\right\},\left\{F\right\}\right\},$$

and integrated cause information

$$\begin{array}{cc} \varphi_{c}\left(m\right) & =\mathrm{ID}(\pi_{c}\left(ABF|ABCD=↑↑↑↓\right),\pi_{c}^{\psi^{*}}\left(ABF|ABCD=↑↑↑↓)\right)\\ \; & =0.09. \end{array}$$

Since M={A,B,C,D}={↑,↑,↑,↓} has an irreducible cause ($\varphi_{c}>0$) and effect ($\varphi_{e}>0$), we say that the mechanism ABCD *exists* within the system with integrated information

$$\varphi \left(m\right)=\min\{\varphi_{e}\left(m\right),\varphi_{c}\left(m\right)\}=0.04.$$

This means that, given the system *S*, the mechanism M={A,B,C,D}⊆S in state $m=\left\{↑,↑,↑,↓\right\}\in \Omega_{M}$ specifies the *distinction*

$$\begin{array}{c} X(\{A,B,C,D\}=\{↑,↑,↑,↓\})\\ \;\;=\left\{(Z_{c}^{*}=\left\{A,B,F\right\}=\left\{↑,↓,↓\right\},Z_{e}^{*}=\left\{A,B,C\right\}=\{↑,↑,↑\},\varphi \left(\left\{A,B,C,D\right\}\right)=0.04)\right\}. \end{array}$$

## 5. Discussion

Mechanism integrated information φ(m) is a measure of the intrinsic cause–effect power of a mechanism M=m within a system. It reflects how much a mechanism as a whole (above and beyond its parts) constrains the units in its cause and effect purview. We characterize three properties of information based on the postulates of IIT: *causality*, *intrinsicality*, and *specificity*, and demonstrate that there is a unique measure (ID) that satisfies these properties. Notably, intrinsicality requires that information increases when expanding a purview with a fully constrained unit (expansion) but decreases when expanding a purview with a fully unconstrained unit (dilution). In situations with partial constraint, finding a unique measure gives us a principled way to balance expansion and dilution.

Early versions of IIT used the KLD to measure the difference between probability distributions [4,16]. The KLD was a practical solution given its unique mathematical properties and ubiquity in information theory; however, there was no principled reason to select it over any other measure. In [3], the KLD was replaced by the EMD, which was an initial attempt to capture the idea of relations among distinctions. The more two distinctions overlap in their purview units and states, the smaller the EMD distance between them; this distance was used as the ground distance to compute the system integrated information (Φ). This aspect of the EMD is now encompassed by including relations as an explicit part of the cause–effect structure, defined in a way that is consistent with the postulates of IIT [10]. The new intrinsic difference measure is the first principled measure based on properties derived from the postulates of IIT. Importantly, ID is shown to be the unique measure that satisfies the three properties—causality, intrinsicality and specificity—the KLD and EMD measures do not satisfy intrinsicality or specificity. See Appendix C for an example of how the different measures change the purview with maximum integrated information.

Furthermore, we define a set of possible partitions of a mechanism and its purview (Ψ(M,Z)), which ensures that the mechanism is destroyed (“distintegrated”) after the partition operation is applied. Previous formulations of mechanism integrated information restricted the set of all possible partitions to bipartitions of a mechanism and its purview but allowed for partitions that do not qualify as “disintegrating” the mechanism (for example, cutting away a single purview unit) [3]. For most mechanisms the minimum information partition $\psi^{*}$ still partitions the mechanism in two parts; exceptions tend to occur if multiple inputs to the same unit counteract each other. The requirement for disintegrating partitions is more consequential, especially for first-order mechanisms (those composed of a single unit). Without this restriction, the $\psi^{*}$ of a first-order mechanism would always be to cut away its weakest purview unit, and the integrated information of the mechanism would then be equal to the information the mechanism specifies about its least constrained purview unit. With the disintegrating partitions, a first-order mechanism must be cut away from its entire purview, reflecting the notion that everything that a first-order mechanism does is irreducible (since it is unified).

The particular partition $\psi^{*}\in \Psi \left(M,Z\right)$ that yields the minimum ID between partitioned and unpartitioned repertoires defines the integrated information of a mechanism over a purview. The balance between expansion and dilution, together with the set of possible partitions, allows us to find the purviews $Z_{c}^{*}$ and $Z_{e}^{*}$ with maximum integrated cause and effect information. Moreover, the ID measure identifies the specific cause state $z_{c}^{*}$ and effect state $z_{e}^{*}$ that maximize the mechanism’s integrated cause and effect information. Finally, the overall integrated information of a mechanism *M* in state *m* is the minimum between its integrated cause and effect information: $\varphi \left(m\right)=\min\{\varphi_{c}\left(m\right),\varphi_{e}\left(m\right)\}$.

Mechanisms that exist within a system (φ(m)>0) specify a distinction (a cause and effect) for the system, and the set of all distinctions and the relations among them define the cause–effect structure of the system [10]. As mentioned above (Section 3.1.5), it is in principle possible that there are multiple solutions for $Z_{c}^{*}=z_{c}^{*}$ or $Z_{e}^{*}=z_{e}^{*}$ for a given mechanism *m* in degenerate systems with symmetries in connectivity and functionality (but note that φ(m) is uniquely defined). However, by the exclusion postulate, distinctions within the cause–effect structure of a conscious system should specify a definite cause and effect, which means that they should specify a definite cause and effect purview in a specific state. As also argued in [17], distinctions that are underdetermined should thus not be included in the cause–effect structure until the tie between purviews or states can be resolved. In physical systems that evolve in time with a certain amount of variability and indeterminism, ties are likely short lived and may typically resolve on a faster scale than the temporal scale of experience.

The principles and arguments applied to mechanism information will need to be extended to relation integrated information and system integrated information, laying the ground work for an updated 4.0 version of the theory. Relations describe how causes and effects overlap in the cause–effect structure, by being over the same units and specifying the same state. Like distinctions, relations exist within the cause–effect structure, and their existence is quantified by an analogous notion of relation integrated information ($\varphi_{r}$). Similarly, the intrinsic existence of a candidate system and its cause–effect structure as a PSC with an experience is quantified by system integrated information (Φ). Both $\varphi_{r}$ and Φ measure the difference made by "cutting apart" the object (relation or system) according to its $\psi^{*}$. As a measure of existence, the difference measures used for $\varphi_{r}$ and Φ must also satisfy the causality, intrinsicality and specificity properties. In the case of Φ, the expansion and dilution properties will need to be adapted to the combinatorial nature of the measure, since adding a single unit to a PSC doubles the number of potential distinctions.

According to IIT, a system is a PSC if its cause–effect structure is maximally irreducible (it is a maximum of system integrated information, Φ). Moreover, if a system is a PSC, then its subjective experience is identical to its cause–effect structure [3]. Since the quantity and quality of consciousness are what they are, the cause–effect structure cannot vary arbitrarily with the chosen measure of intrinsic information. For this reason, a measure of intrinsic information that is based on the postulates and is unique is a critical requirement of the theory.

## Figures and Tables

**Figure 5:**
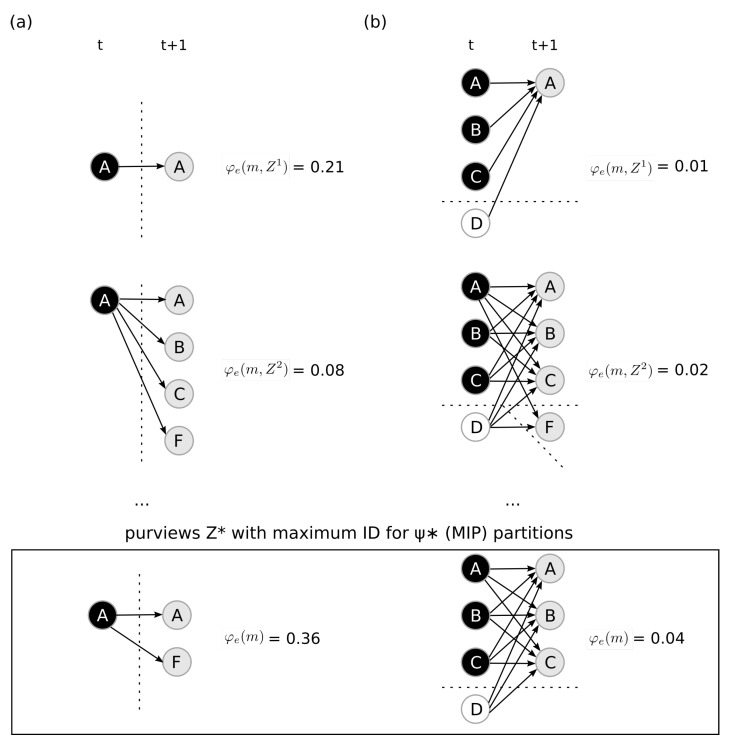
Exclusion. Causal graphs of different mechanisms constraining different purviews. The system *S* used in these examples is the same as in Figure 4a. Each line shows the mechanism *M* constraining different purviews *Z*. (**a**) The mechanism M={A} at state m={↑}. The bottom line shows the purview Z∈S with maximum integrated effect information and the MIP is the complete partition. (**b**) The mechanism M={A,B,C,D} at state m={↑,↑,↑,↓}. The bottom line is the purview Z∈S with maximum integrated effect information and the MIP is $\psi^{*}=\left\{\left(\left\{A,B,C\right\},\left\{A,B,C\right\}\right),\left(\left\{D\right\},\left\{\emptyset \right\}\right)\right\}$.

**Figure A1:**
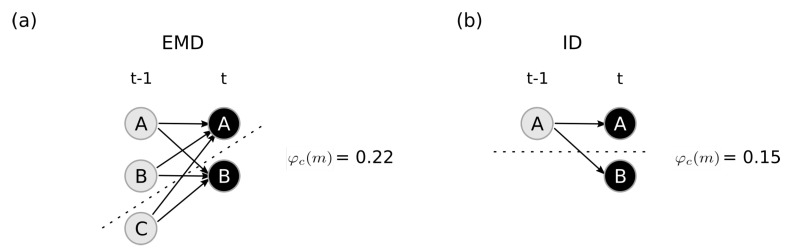
Comparison between earth mover’s distance (EMD) and ID. Using the same system *S* used in Figure 4a, we find the cause purview with maximum integrated information for the mechanism M={A,B} in state m={↑,↑}, which is larger when using the EMD measure (**a**) when compared to the ID measure (**b**). The integrated information when using the EMD measure is also larger than the ID measure.

## Data Availability

No new data were created or analyzed in this study. Data sharing is not applicable to this article.

## Appendix A. Cause and Effect Repertoires

The cause and the effect repertoire can be derived from the system defined in Equation (1). The random variables $S_{i}$ define the system state space $\Omega_{S}=\times_{i=1}^{n}\Omega_{S_{i}}$, where × is the cross product of each individual state space. We also require that the random variables are conditional independent

$$\begin{array}{cc} p(s_{t+1}|s_{t}) & =\prod_{i=1}^{n}p\left(s_{i,t+1}|s_{t}\right), \end{array}$$

that the transitions are time invariant

$$\begin{array}{cc} p(s_{t+1}|s_{t}) & =p(s_{t}|s_{t-1}), \end{array}$$

and that the probabilities are well-defined for all possible states

$$\begin{array}{cc} \;\exists \;\;p(s_{t+1}|s_{t})\; & \;\mathrm{for}\;\mathrm{all}\;\;s_{t},s_{t+1}\in \Omega_{S}. \end{array}$$

Given the former definitions, the stochastic system *S* corresponds to a causal network where $p\left(s_{t+1}|s_{t}\right)=p\left(s_{t+1}|do\left(s_{t}\right)\right)$ [12,18,19].

We use uppercase letters as parameters of the probability function to define probability distributions, e.g., $p\left(S_{t+1}|s_{t}\right)=\left\{p\left(s_{t+1}|s_{t}\right):\;s_{t+1}\in \Omega_{S}\right\}$, and the operators ∑ and ∏ are applied to each state independently.

### Appendix A.1. Causal Marginalization

Given a mechanism M⊆S in a state $m_{t}\in \Omega_{M}$ and a purview Z⊆S, *causal marginalization* serves to remove any contributions to the repertoire of states $\Omega_{Z}$ that are outside the mechanism *M* and purview *Z*. Explicitly, given the set W=S\M, we define the effect repertoire of a single unit $Z_{i}\in Z$ as

$$\pi_{e}\left(Z_{i}|m\right)=\sum_{w_{t}\in \Omega_{W}}p\left(Z_{i,t+1}|m_{t},w_{t}\right){\left|\Omega_{W}\right|}^{-1}.$$

Note that, for causal marginalization, we impose a uniform distribution as $p(W_{t})$. This ensures that the repertoire captures the constraints due to the mechanism alone and not to whatever external factors might bias the variables in *W* to one state or another.

Given the set V=S\Z, the cause repertoire for a single unit $M_{i}\in M$, using Bayes’ rule, is

$$\pi_{c}\left(Z|m_{i}\right)=\frac{\sum_{v_{t-1}\in \Omega_{V}}p\left(m_{i,t}|Z_{t-1},v_{t-1}\right)}{\sum_{s_{t-1}\in \Omega_{S}}p\left(m_{i,t}|s_{t-1}\right)},$$

where again we impose the uniform distributions as $p(V_{t-1})$, $p(Z_{t-1})$, and $p(S_{t-1})$.

Note that the transition probability function $p(Z_{t+1}|m_{t})$ not only contains dependencies of $Z_{t+1}$ on $m_{t}$ but also correlations between the variables in *Z* due to common inputs from units in *W*, which should not be counted as constraints due to $m_{t}$. To discount such correlations, we define the effect repertoire over a set *Z* of *r* units $Z_{i}$ as the product of the effect repertoires over individual units

(A1) $$\pi_{e}\left(Z|m\right)=\overset{r}{\underset{i=1}{⨂}}\pi_{e}\left(Z_{i}|m\right),$$

where ⨂ is the Kronecker product of the probability distributions. In the same manner, given that the mechanism *M* has *q* units $M_{i}$, we define the cause repertoire of *Z* as

(A2) $$\pi_{c}\left(Z|m\right)=\frac{\prod_{i=1}^{q}\pi_{c}\left(Z|m_{i}\right)}{\sum_{z\in \Omega_{Z}}\prod_{i=1}^{q}\pi_{c}\left(z|m_{i}\right)}.$$

### Appendix A.2. Partitioned Repertoires

Given a partition ψ∈Ψ(M,Z) constituted of *k* parts (see Equation (4)), we can define the partitioned repertoire

(A3) $$\pi^{\psi }\left(Z|m\right)=\overset{k}{\underset{j=1}{⨂}}\pi \left(Z_{j}|m_{j}\right),$$

with $\pi \left(\emptyset |m_{j}\right)=\pi \left(\emptyset \right)=1$. In the case of $m_{j}=\emptyset$, $\pi \left(Z_{j}|\emptyset \right)=\pi \left(Z_{j}\right)$ corresponds to an unconstrained effect repertoire

(A4) $$\pi_{e}\left(Z\right)=\overset{r}{\underset{i=1}{⨂}}\pi_{e}\left(Z_{i}\right)=\overset{r}{\underset{i=1}{⨂}}\sum_{s_{t}\in \Omega_{S}}p\left(Z_{i,t+1}|s_{t}\right){\left|\Omega_{S}\right|}^{-1},$$

which follows from Equation (A1) and cause repertoire

(A5) $$\pi_{c}\left(Z\right)=\frac{1}{|\Omega_{Z}|},$$

which follows from Equation (A2).

## Appendix B. Full Statement and Proof of Theorem 1

We now give the full statement and proof of the Theorem 1, demonstrating the uniqueness of the function *f* (see also [15,20]). We start with some preliminary definitions:

$$R^{+}=\left\{x\in R:x>0\right\},\;N_{2}=\left\{2,3,4,\ldots \right\},$$

$$J=\left(0,1\right),\;\hat{J}=\left(0,1\right],\;\bar{J}=\left[0,1\right],\;K=\left(\bar{J}\times \bar{J}\right)\backslash \left(\hat{J}\times \left\{0\right\}\right),$$

$$I_{\delta }\left(x\right)=\left\{y\in J:|x-y|<\delta \right\}.$$

For each $n\in N_{2}$, we further define

$$\Gamma^{n}=\left\{X^{n}=\left(x_{1},\ldots ,x_{n}\right):x_{1},\ldots ,x_{n}\in \bar{J}\;,\;\sum_{\alpha =1}^{n}x_{\alpha }=1\right\},$$

$$V^{n}=\left(1,0,\ldots ,0\right)\in \Gamma^{n},\;U^{n}=\left(\frac{1}{n},\ldots ,\frac{1}{n}\right)\in \Gamma^{n},$$

$$\Delta^{n}=\left\{\left(X^{n},Y^{n}\right)\in \Gamma^{n}\times \Gamma^{n}:\left(x_{\alpha },y_{\alpha }\right)\in K,\;\mathrm{for}\;\mathrm{all}\;\alpha \in \left\{1,\ldots ,n\right\}\right\}.$$

Using these definitions, we further define the following properties.

- **Property I**: *Causality*. Let $\left(P^{n},Q^{n}\right)\in \Delta^{n}$. The difference $D(P^{n},Q^{n})$ is defined as $D:\Delta^{n}\to R$, such that
  $$D\left(P^{n},Q^{n}\right)=0\Leftrightarrow P^{n}=Q^{n}.$$
- **Property II**: *Intrinsicality*. Let $\left(P^{l},Q^{l}\right)\in \Delta^{l}$ and $\left(P^{m},Q^{m}\right)\in \Delta^{m}$. Then
  (a) expansion: $D\left(V^{l}⊗V^{m},P^{l}⊗Q^{m}\right)=D\left(V^{l},P^{l}\right)+D\left(V^{m},Q^{m}\right),$
  (b) dilution: $D\left(P^{l}⊗U^{m},Q^{l}⊗U^{m}\right)=\frac{D\left(P^{l},Q^{l}\right)+D\left(U^{m},U^{m}\right)}{m},$
  where $P^{l}⊗Q^{m}=\left(p_{1}q_{1},\ldots ,p_{1}q_{m},\ldots ,p_{l}q_{1},\ldots ,p_{l}q_{m}\right)\in \Gamma^{lm}$ and from Property I $D(U^{m},U^{m})=0$.
- **Property III**: *Specificity*. The difference must be state-specific, meaning there exists f:K⟶R such that for all $\left(P^{n},Q^{n}\right)\in \Delta^{n}$ we have $D\left(P^{n},Q^{n}\right)=f\left(p_{\alpha },q_{\alpha }\right)$, where α∈{1,…,n}, $p_{\alpha }\in P^{n}$ and $q_{\alpha }\in Q^{n}$. More precisely, we define
  $$D\left(P^{n},Q^{n}\right):=\max_{\alpha }\{|f\left(p_{\alpha },q_{\alpha }\right)\left|\right\},$$
  where f is continuous on K, analytic on $\hat{J}\times J$ and $f(0,q_{\alpha })$ is analytic on J.

The following lemma allows the analytic extension of real analytic functions.

**Lemma** **A1** (See Proposition 1.2.3 in [21])**.** *If f and g are real analytic functions on an open interval U∈R and if there is a sequence of distinct points ${\left\{x_{n}\right\}}_{n}\in U$ with $x_{0}=\lim_{n\to \infty }x_{n}\in U$ such that*

$$f\left(x_{n}\right)=g\left(x_{n}\right),$$

*then*

f(x)=g(x),for allx∈U.

**Corollary** **A1** (See Corollary 1.2.6 in [21])**.** *If f and g are analytic functions on an open interval U and if there is an open interval W⊆U such that*

f(x)=g(x),for allx∈W,

*then*

f(x)=g(x),for allx∈U.

The following lemma shows that a strict maximum over continuous functions, each evaluated at fixed points, must hold for an open interval around such fixed points.

**Lemma** **A2.**
*Let $g_{\alpha }:J\to R$ be continuous functions, where α∈{1,…,n}, fix $x_{1},\ldots ,x_{n}\in J$. If there exists $\alpha^{*}$ such that for $\alpha \neq \alpha^{*}$,*

$$g_{\alpha^{*}}\left(x_{\alpha^{*}}\right)>g_{\alpha }\left(x_{\alpha }\right),$$

*then there exists δ>0 such that*

$$\max_{\alpha }\left\{g_{\alpha }\left({\hat{x}}_{\alpha }\right)\right\}=g_{\alpha^{*}}\left({\hat{x}}_{\alpha^{*}}\right),$$

*for all ${\hat{x}}_{\alpha }\in I_{\delta }\left(x_{\alpha }\right)$ and for all α∈{1,…,n}.*

We now provide the solution to a functional equation similar to the Pexider logarithmic equation [22].

**Lemma** **A3.**
*Let f,g,h:J→R be analytic functions on J. Suppose the functional equation*

|f(pq)|=max{|g(p)|,|h(p)|}+max{|g(q)|,|h(q)|},

*holds for all $pq\in I_{\delta }\left(p^{\prime }q^{\prime }\right)$, where $I_{\delta }\left(p^{\prime }q^{\prime }\right)\subseteq J$. Then there exists c,d∈R such that*

f(x)=clog(x)+d,for allx∈J.

**Proof.** First, for some i∈{g,h} suppose that there exists $(p_{i},q_{i})\in J\times J$ such that $p_{i}q_{i}\in I_{\delta }\left(p^{\prime }q^{\prime }\right)$ and

$$\begin{array}{cc} \left|i(p_{i})\right| & =\max\{|g\left(p_{i}\right)|,|h\left(p_{i}\right)|\} \end{array}$$

is a strict maximum. Then by Lemma A2 there exists $\delta_{p}>0$ such that

(A6) $$\left|i\left(p\right)\right|=\max\left\{|g\left(p\right)|,|h\left(p\right)|\right\},\;\;\mathrm{for}\;\mathrm{all}\;p\in I_{\delta_{p}}\left(p_{i}\right).$$

Second, if there does not exist $(p_{i},q_{i})\in J\times J$ such that $p_{i}q_{i}\in I_{\delta }\left(p^{\prime }q^{\prime }\right)$ and $\left|i(p_{i})\right|$ is a strict maximum, then we set $q_{i}=q^{\prime }$, $p_{i}=p^{\prime }$ and $\delta_{p}=\frac{\delta }{q^{\prime }}$ so that Equation (A6) holds since |g(p)|=|h(p)| for all (p,q)∈J×J such that $pq\in I_{\delta }\left(p^{\prime }q^{\prime }\right)$. Next, define $\delta^{\prime }:=\min\{\delta -|p_{i}q_{i}-p^{\prime }q^{\prime }\left|,\delta_{p}q^{\prime }\right\}$. Suppose that there exists $q_{j}\in J$ such that $p_{i}q_{j}\in I_{\delta^{\prime }}\left(p_{i}q_{i}\right)$, and for some j∈{g,h},

$$\begin{array}{cc} \left|j(q_{j})\right| & =\max\{|g\left(q_{j}\right)|,|h\left(q_{j}\right)|\} \end{array}$$

is a strict maximum. Then by Lemma A2 there exists $\delta_{q}>0$ such that

(A7) $$\left|j\left(q\right)\right|=\max\left\{|g\left(q\right)|,|h\left(q\right)|\right\},\;\;\mathrm{for}\;\mathrm{all}\;q\in I_{\delta_{q}}\left(q_{j}\right).$$

Finally, if there does not exist $q_{j}\in J$ such that $p_{i}q_{j}\in I_{\delta^{\prime }}\left(p_{i}q_{i}\right)$ and $\left|j(q_{j})\right|$ is a strict maximum, then we set $q_{j}=q_{i}$ and $\delta_{q}=\frac{\delta^{\prime }}{p_{i}}$ so that Equation (A7) holds since |g(q)|=|h(q)| for all (p,q)∈J×J such that $pq\in I_{\delta^{\prime }}\left(p_{i}q_{i}\right)$. Let pq=x and define $\delta^{\prime \prime }:=\min\{\delta^{\prime }-|p_{i}q_{j}-p_{i}q_{i}\left|,\delta_{q}p_{i}\right\}$, then

$$\left|f\left(x\right)\right|=\left|i\left(p\right)\right|+|j\left(q\right)|,\;\;\mathrm{for}\;\mathrm{all}\;x\in I_{\delta^{\prime \prime }}\left(p_{i}q_{j}\right).$$

Moreover, it follows that one of the following options must be true

$$f\left(x\right)=\pm i\left(p\right)\pm j\left(q\right),\;\;\mathrm{for}\;\mathrm{all}\;x\in I_{\delta^{\prime }}\left(p_{i}q_{j}\right).$$

Since the functions are analytic on *J* and therefore twice differentiable, then

$$\frac{\partial }{\partial q}\left[\frac{\partial }{\partial p}\left[f\left(x\right)\right]\right]=x\frac{d}{dx^{2}}f\left(x\right)+\frac{d}{dx}f\left(x\right)=\pm \frac{\partial }{\partial q}\left[\frac{\partial }{\partial p}i\left(p\right)\right]\pm \frac{\partial }{\partial q}\left[\frac{\partial }{\partial p}j\left(q\right)\right]=0.$$

Integrating with respect to *x* yields

f(x)=clog(x)+d,

for c,d∈R and for all $x\in I_{\delta^{\prime \prime }}\left(x^{\prime }\right)$ where $x^{\prime }=p^{\prime }q^{\prime }$. Since *f* is analytic on *J* and since $I_{\delta^{\prime \prime }}\left(x^{\prime }\right)\subset J$, by Corollary A1, we can extend f(x) such that

f(x)=clog(x)+d,for allx∈J.

□

**Lemma** **A4.**
*If $D:\Delta^{n}\to R$ satisfies properties I and III for some f:K⟶R, then*

$$f\left(p,p\right)=0,\;\;for\;all\;p\in \bar{J}.$$

**Proof.** Let $P^{2}=\left(p,1-p\right)\in \Gamma^{2}$. By properties I and III

(A8) $$0=D\left(P^{2},P^{2}\right)=\max\left\{|f(p,p)|,|f(1-p,1-p)|\right\},\;\;\mathrm{for}\;\mathrm{all}\;p\in \bar{J}.$$

□

**Theorem** **A1.**
*Let $\left(P^{n},Q^{n}\right)\in \Delta^{n}$ for some $n\in N_{2}$ and $D:\Delta^{n}\to R$ where D satisfies properties I, II and III. Then*

(A9) $$D\left(P^{n},Q^{n}\right)=\max_{\alpha }\{|f\left(p_{\alpha },q_{\alpha }\right)\left|\right\},$$

*where for some k∈R\{0},*

(A10) $$f\left(p,q\right)=k\;p\log\left(\frac{p}{q}\right),\;\;for\;all\;\left(p,q\right)\in K.$$

**Proof** **of** **Theorem** **A1.** First we show that the function in Equation (A10) satisfies properties I, II and III. To see that the function satisfies Property I, notice that for each $\left(P^{n},Q^{n}\right)\in \Delta^{n}$ where $P^{n}\neq Q^{n}$, since k≠0, then there exists β∈{1,…,n} such that

$$D\left(P^{n},Q^{n}\right)=\max_{\alpha }\left\{\left|kp_{\alpha }\log\left(\frac{p_{\alpha }}{q_{\alpha }}\right)\right|\right\}\geq \left|kp_{\beta }\log\left(\frac{p_{\beta }}{q_{\beta }}\right)\right|>0,$$

and for each $P^{n}=Q^{n}$,

$$D\left(P^{n},P^{n}\right)=\max_{\alpha }\left\{\left|kp_{\alpha }\log\left(\frac{p_{\alpha }}{p_{\alpha }}\right)\right|\right\}=0.$$

To see that it satisfies Property II.a, for each $P^{l}\in \Gamma^{l}$ and for each $Q^{m}\in \Gamma^{m}$

$$\begin{array}{cc} D(V^{l}⊗V^{m},P^{l}⊗Q^{m}) & =\max\left\{\left|k\log\left(\frac{1}{p_{1}q_{1}}\right)\right|,0\right\}=\left|k\log\left(\frac{1}{p_{1}q_{1}}\right)\right|\\ \; & =\left|k\log\left(\frac{1}{p_{1}}\right)\right|+\left|k\log\left(\frac{1}{q_{1}}\right)\right|\\ \; & =\max\left\{\left|k\log\left(\frac{1}{p_{1}}\right)\right|,0\right\}+\max\left\{\left|k\log\left(\frac{1}{q_{1}}\right)\right|,0\right\}\\ \; & =D\left(V^{l},P^{l}\right)+D\left(V^{m},Q^{m}\right). \end{array}$$

Similarly by Property II.b notice that for each $\left(P^{l},Q^{l}\right)\in \Delta^{l}$

$$\begin{array}{cc} D(P^{l}⊗U^{m},Q^{l}⊗U^{m}) & =\max_{\alpha }\left\{\left|\frac{kp_{\alpha }}{m}\log\left(\frac{p_{\alpha }}{q_{\alpha }}\right)\right|\right\}\\ \; & =\frac{1}{m}\max_{\alpha }\left\{\left|kp_{\alpha }\log\left(\frac{p_{\alpha }}{q_{\alpha }}\right)\right|\right\}\\ \; & =\frac{1}{m}D\left(P^{l},Q^{l}\right). \end{array}$$

It is clear that the function *f* in Equation (A10) satisfies Property III.

The remaining part of the proof is divided into two steps:

**Step 1.** First we show that under four assumptions, *f* satisfies properties I, II and III iff $f\left(p,q\right)=kp\log\left(\frac{p}{q}\right),\;k\in R\backslash \left\{0\right\}$.

**Step 2.** Next we show that if any of our assumptions is violated, then no suitable *f* exists.

**Verification of Step 1.** We apply Property II.a with $P^{2}=\left(p,1-p\right),Q^{2}=\left(q,1-q\right)$ for some p,q∈J where p≠q. We then have

$$\begin{array}{cc} D(V^{2}⊗V^{2},P^{2}⊗Q^{2}) & =D\left(V^{2},P^{2}\right)+D\left(V^{2},Q^{2}\right). \end{array}$$

By Property III, the following identity holds

(A11) $$\begin{array}{c} \begin{array}{c} \max\left\{|f(1,pq)|,|f(0,p(1-q))|,|f(0,(1-p)q)|,|f(0,(1-p)(1-q))|\right\}\\ =\max\left\{|f(1,p)|,|f(0,1-p)|\right\}+\max\left\{|f(1,q)|,|f(0,1-q)|\right\}. \end{array} \end{array}$$

Our first assumption (AS1) states that there exists some $p^{\prime },q^{\prime }\in J$ such that $\left|f(1,p^{\prime }q^{\prime })\right|$ is a strict maximum. By Lemma A2, there exists a δ>0 such that

(A12) $$\begin{array}{c} \left|f\left(1,pq\right)\right|=\max\left\{|f(1,p)|,|f(0,1-p)|\right\}+\max\left\{|f(1,q)|,|f(0,1-q)|\right\}, \end{array}$$

for all $pq\in I_{\delta }\left(p^{\prime }q^{\prime }\right)$. Further, by Lemma A3 there exists c,d∈R such that

f(1,q)=clog(q)+d,for allq∈J,

and since by Property III *f* is continuous, the application of Lemma A4 yields

$$\lim_{q\to 1}f\left(1,q\right)=\lim_{q\to 1}c\log\left(q\right)+d=d=0,$$

i.e., for $k_{1}=-c$, we have

(A13) $$f\left(1,q\right)=k_{1}\log\left(\frac{1}{q}\right),\;\;\mathrm{for}\;\mathrm{all}\;q\in J.$$

Now applying Property II.b for l=2, $P^{2}=V^{2}$, $Q^{2}=\left(r,1-r\right)$ for all r∈J and for each $m\in N_{2}$, we have

$$\begin{array}{c} D\left(V^{2}⊗U^{m},Q^{2}⊗U^{m}\right)=\frac{1}{m}D\left(V^{2},Q^{2}\right), \end{array}$$

and by Property III

(A14) $$\begin{array}{c} \max\left\{\left|f\left(\frac{1}{m},\frac{r}{m}\right)\right|,\left|f\left(0,\frac{1-r}{m}\right)\right|\right\}=\frac{1}{m}\max\left\{|f(1,r)|,|f(0,1-r)|\right\}, \end{array}$$

for all r∈J. Our second assumption (AS2) states that there exists $q_{1}\in J$ such that $|f\left(1,q_{1}\right)|>|f\left(0,1-q_{1}\right)|$. For some a∈{−1,1}, we have

$$\max\{|f\left(1,q_{1}\right)|,|f\left(0,1-q_{1}\right)\left|\right\}=af\left(1,q_{1}\right).$$

Further, by Lemma A2 and Equation (A13), there exists δ>0 such that

$$\max\left\{|f\left(1,r\right)|,|f\left(0,1-r\right)|\right\}=af\left(1,r\right)=ak_{1}\log\left(\frac{1}{r}\right),\;\;\mathrm{for}\;\mathrm{all}\;r\in I_{\delta }\left(q_{1}\right).$$

Plugging this result back into Equation (A14) yields

(A15) $$\max\left\{\left|f\left(\frac{1}{m},\frac{r}{m}\right)\right|,\left|f\left(0,\frac{1-r}{m}\right)\right|\right\}=\frac{ak_{1}}{m}\log\left(\frac{1}{r}\right),\;\;\mathrm{for}\;\mathrm{all}\;r\in I_{\delta }\left(q_{1}\right).$$

Our third assumption (AS3) states that $\left|f\left(0,\frac{1-r}{m}\right)\right|$ is never a strict maximum in Equation (A15), so that for some b∈{−1,1}, we have

$$\begin{array}{c} \max\left\{\left|f\left(\frac{1}{m},\frac{r}{m}\right)\right|,\left|f\left(0,\frac{1-r}{m}\right)\right|\right\}=bf\left(\frac{1}{m},\frac{r}{m}\right),\;\;\mathrm{for}\;\mathrm{all}\;r\in I_{\delta }\left(q_{1}\right). \end{array}$$

Let $q=\frac{r}{m}$ and let $I=I_{\frac{\delta }{m}}\left(\frac{r}{m}\right)\cap I_{\frac{\delta }{m}}\left(\frac{q_{1}}{m}\right)$. Then by Equation (A15)

$$\begin{array}{c} f\left(\frac{1}{m},q\right)=\frac{k_{m}}{m}\log\left(\frac{1}{mq}\right),\;\;\mathrm{for}\;\mathrm{all}\;q\in I, \end{array}$$

where $k_{m}=\frac{ak_{1}}{b}$. By Corollary A1, we can extend $f\left(\frac{1}{m},q\right)$ to *J*

(A16) $$f\left(\frac{1}{m},q\right)=\frac{k_{m}}{m}\log\left(\frac{1}{mq}\right),\;\;\;\mathrm{for}\;\mathrm{all}\;q\in J,$$

where $k_{m}\in \left\{+k_{1},-k_{1}\right\}$. Let $n\in N_{2}$ and let $0<q_{2}<\frac{n-1}{2n}$, then $q_{2}\in J$. By Property II.b for l=2, $P^{2}=\left(\frac{n-1}{2n},\frac{n+1}{2n}\right)$, $Q^{2}=\left(q_{2},1-q_{2}\right)$ and m=(n−1)(n+1), we have

$$\begin{array}{c} D\left(P^{2}⊗U^{m},Q^{2}⊗U^{m}\right)=\frac{1}{m}D\left(P^{2},Q^{2}\right), \end{array}$$

and by Property III

$$\begin{array}{c} \max\left\{\left|f\left(\frac{1}{2n(n+1)},\frac{q_{2}}{\left(n-1)(n+1\right)}\right)\right|,\left|f\left(\frac{1}{2n(n-1)},\frac{1-q_{2}}{\left(n-1)(n+1\right)}\right)\right|\right\}\\ \;\;\;\;\;=\frac{1}{\left(n-1)(n+1\right)}\max\left\{\left|f\left(\frac{n-1}{2n},q_{2}\right)\right|,\left|f\left(\frac{n+1}{2n},1-q_{2}\right)\right|\right\}. \end{array}$$

By Equation (A16), we have

$$\begin{array}{c} \max\left\{\left|\frac{k_{2n(n+1)}}{2n(n+1)}\log\left(\frac{n-1}{2nq_{2}}\right)\right|,\left|\frac{k_{2n(n-1)}}{2n(n-1)}\log\left(\frac{n+1}{2n(1-q_{2})}\right)\right|\right\}\\ \;\;\;\;\;\;\;\;\;=\frac{1}{\left(n-1)(n+1\right)}\max\left\{\left|f\left(\frac{n-1}{2n},q_{2}\right)\right|,\left|f\left(\frac{n+1}{2n},1-q_{2}\right)\right|\right\}. \end{array}$$

Since $q_{2}<\frac{n-1}{2n}<\frac{1}{2}$, this yields

$$\begin{array}{c} \begin{array}{c} \frac{ak_{2n(n+1)}\left(n-1\right)}{2n}\log\left(\frac{n-1}{2nq_{2}}\right)=\max\left\{\left|f\left(\frac{n-1}{2n},q_{2}\right)\right|,\left|f\left(\frac{n+1}{2n},1-q_{2}\right)\right|\right\}, \end{array} \end{array}$$

for some a∈{+1,−1}. Then we have that for the sequence $h_{\frac{1}{2}}:={\left\{\frac{n-1}{2n}\right\}}_{n}$

(A17) $$\begin{array}{c} \begin{array}{c} k_{p_{n}}p_{n}\log\left(\frac{p_{n}}{q}\right)=\max\left\{|f\left(p_{n},q\right)|,|f\left(1-p_{n},1-q\right)|\right\}, \end{array} \end{array}$$

for all $\left(p_{n},q\right)\in h_{\frac{1}{2}}\times \left(0,p_{n}\right)$, where $k_{p_{n}}=a_{p_{n}}k_{2n(n+1)}$. Our fourth and last assumption (AS4) is that $\left|f(1-p_{n},1-q)\right|$ is a strict maximum only for a finite number of $p_{n}\in h_{\frac{1}{2}}$. More specifically, let

$$\bar{P}=\{p_{n}\in h_{\frac{1}{2}}:\;\exists \;\;q\in \left(0,p_{n}\right)\;s.t.\;|f\left(1-p_{n},1-q\right)|>|f\left(p_{n},q\right)\left|\right\}.$$

Then (A4) states that $\sup\left\{\bar{P}\right\}<\frac{1}{2}$ where, for convention, $\sup\{\bar{P}\}:=-\infty$ if $\bar{P}=\emptyset$. Let $n^{\prime }:=0$ if $\bar{P}=\emptyset$, else there exists $n^{\prime }\in N_{2}$ such that $\sup\left\{\bar{P}\right\}=\frac{n^{\prime }-1}{2n^{\prime }}$. Define $h_{\frac{1}{2}}^{\prime }={\left\{\frac{n+n^{\prime }-1}{2(n+n^{\prime })}\right\}}_{n}$. Then for a fixed $p_{n}\in h_{\frac{1}{2}}^{\prime }$, there exists $q_{n}\in \left(0,p_{n}\right)$ such that

$$\max\left\{bf\left(p_{n},q_{n}\right),\left|f\left(1-p_{n},1-q_{n}\right)\right|\right\}=bf\left(p_{n},q_{n}\right),$$

for some b∈{+1,−1}. By Lemma A2, there exists δ>0 such that

$$\max\left\{bf\left(p_{n},q\right),\left|f\left(1-p_{n},1-q\right)\right|\right\}=bf\left(p_{n},q\right),\;\;\mathrm{for}\;\mathrm{all}\;q\in I_{\delta }\left(q_{n}\right).$$

By Equation (A17), for $I_{n}=\left(0,p_{n}\right)\cap I_{\delta }\left(q_{n}\right)$, we have

(A18) $$f\left(p_{n},q\right)=k_{p_{n}}p_{n}\log\left(\frac{p_{n}}{q}\right),\;\;\mathrm{for}\;\mathrm{all}\;\left(p_{n},q\right)\in h_{\frac{1}{2}}\times I_{n},$$

where the sign *b* was absorbed by the constant $k_{p_{n}}$. By Corollary A1, for a fixed $p_{n}^{*}\in h_{\frac{1}{2}}$, we can extend $f(p_{n}^{*},q)$ to J, i.e.,

$$f\left(p_{n}^{*},q\right)=k_{p_{n}^{*}}p_{n}^{*}\log\left(\frac{p_{n}^{*}}{q}\right),\;\;\mathrm{for}\;\mathrm{all}\;q\in J.$$

For a fixed $q^{*}\in J$, since by Property III *f* is continuous, we have

$$\lim_{n\to \infty }k_{p_{n}}=k\in \left\{+k_{1},-k_{1}\right\}.$$

By Lemma A1, we can uniquely extend $f(p_{n},q^{*})$ to *J* such that

$$f\left(p,q^{*}\right)=kp\log\left(\frac{p}{q^{*}}\right),\;\;\mathrm{for}\;\mathrm{all}\;p\in J.$$

Since this is true for all $q^{*}\in J$, we have that

$$f\left(p,q\right)=kp\log\left(\frac{p}{q}\right),\;\;\mathrm{for}\;\mathrm{all}\;\left(p,q\right)\in \left(J\times J\right).$$

Note that k=0 violates Property I since for some q∈J and $Q^{2}=\left(q,1-q\right)\neq V^{2}$, we have

$$D\left(V^{2},Q^{2}\right)=\max\{|f\left(1,q\right)|,|f\left(0,1-q\right)\left|\right\}=0.$$

By Property III, *f* is continuous in *K* and the following limits exist for all p,q∈J:

$$\begin{array}{cc} f(1,q) & =\lim_{p\to 1^{-}}kp\log\left(\frac{p}{q}\right)=k\log\left(\frac{1}{q}\right),\\ f(p,1) & =\lim_{q\to 1^{-}}kp\log\left(\frac{p}{q}\right)=kp\log\left(p\right),\\ f(0,q) & =\lim_{p\to 0^{+}}kp\log\left(\frac{p}{q}\right)=0,\\ f(0,1) & =\lim_{p\to 0^{+}}kp\log\left(p\right)=0,\\ f(0,0) & =\lim_{p\to 0^{+}}kp\log\left(\frac{p}{p}\right)=0,\\ f(1,1) & =\lim_{p\to 1^{-}}kp\log\left(\frac{p}{p}\right)=0. \end{array}$$

Consequently,

$$f\left(p,q\right)=kp\log\left(\frac{p}{q}\right),\;\;\mathrm{for}\;\mathrm{all}\;\left(p,q\right)\in K.$$

**Verification of Step 2.** Up until here we have showed that Equation (A10) not only defines a function which satisfies properties I, II and III, but it also defines the *only* function which satisfies properties I, II and III for l=m=2 given the following assumptions

AS1: $\;\exists \;p^{\prime },q^{\prime }\in J$ such that $\left|f(1,p^{\prime }q^{\prime })\right|$ is a strict maximum in Equation (A11),
AS2: $\;\exists \;q_{1}\in J$ such that $|f\left(1,q_{1}\right)|>|f\left(0,1-q_{1}\right)|$ in Equation (A14),
AS3: $\left|f\left(0,\frac{1-r}{m}\right)\right|$ is never a strict maximum in Equation (A15),
AS4: $\sup\left\{\bar{P}\right\}<\frac{1}{2}$.

All that is left to prove the theorem is to show that violating any of these assumptions also violates some property. First assume that (A1), (A2) and (A3) are true but (A4) is violated, i.e., $\sup\left\{\bar{P}\right\}=\lim_{n\to \infty }p_{n}=\frac{1}{2}$ for $p_{n}\in h_{\frac{1}{2}}$. Let $p_{n}^{\prime }=1-p_{n}$, $q^{\prime }=1-q$ and let $g={\left\{1-p_{i}\right\}}_{i}$ be the sequence of ordered elements in $\bar{P}$ such that $p_{i}<p_{i+1}$. Then by Equation (A17), for all $p_{n}^{\prime }\in g$, there exists $q^{\prime }\in \left(p_{n}^{\prime },1\right)$ such that

$$f\left(p_{n}^{\prime },q^{\prime }\right)=k_{p_{n^{\prime }}}\left(1-p_{n}^{\prime }\right)\log\left(\frac{1-p_{n}^{\prime }}{1-q^{\prime }}\right).$$

By Lemma A2, for a fixed $p_{n}^{*}\in g$, there exists $\delta^{\prime }>0$ such that

$$f\left(p_{n}^{*},q\right)=k_{p_{n}^{*}}\left(1-p_{n}^{*}\right)\log\left(\frac{1-p_{n}^{*}}{1-q}\right),\;\;\mathrm{for}\;\mathrm{all}\;q\in I_{\delta^{\prime }}\left(q^{\prime }\right).$$

Since this holds for all $p_{n}^{*}\in g$, we have

$$f\left(p_{n}^{\prime },q\right)=k_{p_{n^{\prime }}}\left(1-p_{n}^{\prime }\right)\log\left(\frac{1-p_{n}^{\prime }}{1-q}\right),\;\;\mathrm{for}\;\mathrm{all}\;\left(p_{n}^{\prime },q\right)\in g\times I_{\delta^{\prime }}\left(q^{\prime }\right).$$

Similarly to Equation (A18), using the sequence g instead of the sequence $h_{\frac{1}{2}}$, this result can be extended to J×J, i.e.,

(A19) $$f\left(p,q\right)=k\left(1-p\right)\log\left(\frac{1-p}{1-q}\right),\;\;\mathrm{for}\;\mathrm{all}\;\left(p,q\right)\in \left(J\times J\right).$$

Applying Property II.b to l=m=2, $P^{2}=\left(p,1-p\right)$, $Q^{2}=\left(q,1-q\right)$ with q≠p yields

$$D\left(P^{2}*U^{2},Q^{2}*U^{2}\right)=\frac{1}{2}D\left(P^{2},Q^{2}\right).$$

Further, by Property III

$$\max\left\{\left|f\left(\frac{p}{2},\frac{q}{2}\right)\right|,\left|f\left(\frac{1-p}{2},\frac{1-q}{2}\right)\right|\right\}=\frac{1}{2}\max\left\{\left|f(p,q)\right|,\left|f(1-p,1-q)\right|\right\}.$$

However this contradicts Equation (A19) since

$$\begin{array}{c} \max\left\{\left|k\left(1-\frac{p}{2}\right)\log\left(\frac{1-\frac{p}{2}}{1-\frac{q}{2}}\right)\right|,\left|k\left(1-\frac{1-p}{2}\right)\log\left(\frac{1-\frac{1-p}{2}}{1-\frac{1-q}{2}}\right)\right|\right\}\\ \;\;\;\;\;\;\;\;\;\;\;\;\;\;\;\neq \frac{1}{2}\max\left\{\left|k\left(1-p\right)\log\left(\frac{1-p}{1-q}\right)\right|,\left|kp\log\left(\frac{p}{q}\right)\right|\right\}. \end{array}$$

Next we assume that (AS1) and (AS2) are true but (AS3) is violated, meaning that there exists $\left(m^{\prime },r^{\prime }\right)\in N_{2}\times I_{\delta }\left(q_{1}\right)$ such that $|f\left(0,\frac{1-r}{m}\right)|$ is a strict maximum in Equation (A15), i.e.,

$$\begin{array}{c} f\left(0,\frac{1-r^{\prime }}{m^{\prime }}\right)=\frac{ak_{1}}{m^{\prime }}\log\left(\frac{1}{r^{\prime }}\right), \end{array}$$

for some a∈{−1,1}. For some $q^{\prime }=\frac{1-r^{\prime }}{m^{\prime }}\in I_{\frac{\delta }{m^{\prime }}}\left(\frac{1-q_{1}}{m^{\prime }}\right)$, we have

$$\begin{array}{c} f\left(0,q^{\prime }\right)=\frac{ak_{1}}{m^{\prime }}\log\left(\frac{1}{1-m^{\prime }q^{\prime }}\right). \end{array}$$

By Lemma A2, there exists $\delta^{\prime }>0$ such that

$$\begin{array}{c} f\left(0,q\right)=\frac{ak_{1}}{m^{\prime }}\log\left(\frac{1}{1-m^{\prime }q}\right),\;\;\mathrm{for}\;\mathrm{all}\;q\in I_{\delta^{\prime }}\left(q^{\prime }\right). \end{array}$$

By Corollary A1, we can extend $f\left(0,q\right)$ to $\left(0,\frac{1}{m^{\prime }}\right)$, i.e.,

$$f\left(0,q\right)=\frac{ak_{1}}{m^{\prime }}\log\left(\frac{1}{1-m^{\prime }q}\right),\;\;\mathrm{for}\;\mathrm{all}\;q\in \left(0,\frac{1}{m^{\prime }}\right).$$

However, this implies that *f* is discontinuous and violates Property III since

$$\lim_{q\to \frac{1}{m^{\prime }}}f\left(0,q\right)=\lim_{q\to \frac{1}{m^{\prime }}}\frac{ak_{1}}{m^{\prime }}\log\left(\frac{1}{1-m^{\prime }q}\right)=\pm \infty .$$

We now assume that AS1 is true but AS2 is violated, i.e.,

|f(1,q)|≤|f(0,1−q)|,for allq∈J.

For some $p^{\prime },q^{\prime }\in J$ and δ>0, by Equation (A11) and by Equation (A13)

(A20) $$\begin{array}{c} \left|k_{1}\log\left(\frac{1}{pq}\right)\right|=\left|f\left(0,1-p\right)\right|+|f\left(0,1-q\right)|,\;\;\mathrm{for}\;\mathrm{all}\;pq\in I_{\delta }\left(p^{\prime }q^{\prime }\right). \end{array}$$

Let $q_{1},q_{2}\in J$ and let $p^{\prime }q_{1},p^{\prime }q_{2}\in I_{\delta^{\prime }}\left(p^{\prime }q^{\prime }\right)$, then

$$\begin{array}{c} F\left(q_{1}\right)=\left|k_{1}\log\left(\frac{1}{q_{1}}\right)\right|-|f\left(0,1-q_{1}\right)|=|f\left(0,1-p^{\prime }\right)|-\left|k_{1}\log\left(\frac{1}{p^{\prime }}\right)\right|\\ =\left|k_{1}\log\left(\frac{1}{q_{2}}\right)\right|-\left|f\left(0,1-q_{2}\right)\right|=F\left(q_{2}\right). \end{array}$$

Therefore, F(q)=d is constant and

$$\begin{array}{c} \left|f\left(0,1-q\right)\right|=\left|k_{1}\log\left(\frac{1}{q}\right)\right|+d,\;\;\mathrm{for}\;\mathrm{all}\;q\in I_{\frac{\delta }{p^{\prime }}}\left(q^{\prime }\right). \end{array}$$

Plugging this back into Equation (A20) we see that d=0, and for some a∈{−1,1}, there exists $q^{*}\in I_{\frac{\delta }{p^{\prime }}}\left(q^{\prime }\right)$ such that

$$\begin{array}{c} f\left(0,q^{*}\right)=ak_{1}\log\left(\frac{1}{1-q^{*}}\right). \end{array}$$

By Lemma A2, there exists $\delta^{\prime }>0$ such that

$$\begin{array}{c} f\left(0,q\right)=ak_{1}\log\left(\frac{1}{1-q}\right),\;\;\mathrm{for}\;\mathrm{all}\;q\in I_{\delta^{\prime }}\left(q^{*}\right), \end{array}$$

and by Corollary A1

$$\begin{array}{c} f\left(0,q\right)=ak_{1}\log\left(\frac{1}{1-q}\right),\;\;\mathrm{for}\;\mathrm{all}\;q\in J. \end{array}$$

However this is a contradiction since by Equation (A13), for $k_{1}\neq 0$ and $x<\frac{1}{2}$, we have

$$\left|f\left(1,x\right)\right|=\left|k_{1}\log\left(\frac{1}{x}\right)\right|>\left|k_{1}\log\left(\frac{1}{1-x}\right)\right|=\left|f\left(0,1-x\right)\right|.$$

Note that $k_{1}=0$ violates Property I since for any q∈J and $Q^{2}=\left(q,1-q\right)\neq V^{2}$, we have

$$D\left(V^{2},Q^{2}\right)=\max\left\{\left|k_{1}\log\left(\frac{1}{q}\right)\right|,\left|k_{1}\log\left(\frac{1}{1-q}\right)\right|\right\}=0.$$

Finally, if AS1 is violated then there does not exist $p^{\prime },q^{\prime }\in J$ such that $\left|f(1,p^{\prime }q^{\prime })\right|$ is a strict maximum in Equation (A11). Hence, for all p,q∈J, there exists $x^{\prime }\in \left\{p\left(1-q\right),q\left(1-p\right),\left(1-p\right)\left(1-q\right)\right\}$ such that

$$|f\left(0,x^{\prime }\right)|=\max\left\{|f(1,p)|,|f(0,1-p)|\right\}+\max\left\{|f(1,q)|,|f(0,1-q)|\right\}.$$

If $\left|f(0,x^{\prime })\right|$ is a strict maximum, then by Lemma A2 there exists δ>0 such that

(A21) $$\left|f\left(0,x\right)\right|=\max\left\{|f(1,p)|,|f(0,1-p)|\right\}+\max\left\{|f(1,q)|,|f(0,1-q)|\right\},$$

for all $x\in I_{\delta }\left(x^{\prime }\right)$. If there does not exist $x^{\prime }\in \left\{p\left(1-q\right),q\left(1-p\right),\left(1-p\right)\left(1-q\right)\right\}$ such that $\left|f(0,x^{\prime })\right|$ is a strict maximum, then there must exist x∈{p(1−q),q(1−p),(1−p)(1−q)} such that Equation (A21) holds for all x∈J. In both cases, by Lemma A3 there exists c,d∈R such that

f(0,x)=clog(x)+d,for allx∈J.

However, this implies that *f* is discontinuous since

$$\lim_{x\to 0}f\left(0,x\right)=\lim_{x\to 0}c\log\left(x\right)+d=\pm \infty ,$$

even though f(0,0)=0 by Lemma A4. □

## Appendix C. Comparison between ID and EMD

Since the different information measures satisfy different properties, the distinctions that exist in a given system may be different depending on the information measure used to compute integrated information. Here, using the same system used in Figure 5, we provide an example where the cause purview with maximum integrated information is larger when using the EMD measure (Figure A1a) when compared to the same mechanism when using the ID measure (Figure A1b).
